# A Silent Operon of Photorhabdus luminescens Encodes a Prodrug Mimic of GTP

**DOI:** 10.1128/mbio.00700-22

**Published:** 2022-05-16

**Authors:** Negar Shahsavari, Boyuan Wang, Yu Imai, Miho Mori, Sangkeun Son, Libang Liang, Nils Böhringer, Sylvie Manuse, Michael F. Gates, Madeleine Morrissette, Rachel Corsetti, Josh L. Espinoza, Chris L. Dupont, Michael T. Laub, Kim Lewis

**Affiliations:** a Antimicrobial Discovery Center, Department of Biology, Northeastern University, Boston, Massachusetts, USA; b Department of Pharmacology, University of Texas Southwestern Medical Center, Dallas, Texas, USA; c Department of Biology, Massachusetts Institute of Technology, Cambridge, Massachusetts, USA; d Institute for Insect Biotechnology, Justus-Liebig-University of Giessen, Giessen, Germany; e German Center of Infection Research (DZIF), Partner Site Gießen-Marburg-Langen, Gießen, Germany; f J. Craig Venture Institute, La Jolla, California, USA; g Howard Hughes Medical Institute, Massachusetts Institute of Technology, Cambridge, Massachusetts, USA; McMaster University

**Keywords:** antibiotic resistance, natural product, nucleoside analog

## Abstract

With the overmining of actinomycetes for compounds acting against Gram-negative pathogens, recent efforts to discover novel antibiotics have been focused on other groups of bacteria. Teixobactin, the first antibiotic without detectable resistance that binds lipid II, comes from an uncultured *Eleftheria terra*, a betaproteobacterium; odilorhabdins, from *Xenorhabdus*, are broad-spectrum inhibitors of protein synthesis, and darobactins from *Photorhabdus* target BamA, the essential chaperone of the outer membrane of Gram-negative bacteria. *Xenorhabdus* and *Photorhabdus* are symbionts of the nematode gut microbiome and attractive producers of secondary metabolites. Only small portions of their biosynthetic gene clusters (BGC) are expressed *in vitro.* To access their silent operons, we first separated extracts from a small library of isolates into fractions, resulting in 200-fold concentrated material, and then screened them for antimicrobial activity. This resulted in a hit with selective activity against Escherichia coli, which we identified as a novel natural product antibiotic, 3′-amino 3′-deoxyguanosine (ADG). Mutants resistant to ADG mapped to *gsk* and *gmk*, kinases of guanosine. Biochemical analysis shows that ADG is a prodrug that is converted into an active ADG triphosphate (ADG-TP), a mimic of GTP. ADG incorporates into a growing RNA chain, interrupting transcription, and inhibits cell division, apparently by interfering with the GTPase activity of FtsZ. Gsk of the purine salvage pathway, which is the first kinase in the sequential phosphorylation of ADG, is restricted to E. coli and closely related species, explaining the selectivity of the compound. There are probably numerous targets of ADG-TP among GTP-dependent proteins. The discovery of ADG expands our knowledge of prodrugs, which are rare among natural compounds.

## INTRODUCTION

The antimicrobial resistance crisis is caused primarily by multidrug-resistant bacteria such as Escherichia coli, Pseudomonas aeruginosa, and Acinetobacter baumannii (1). This shift in significance, especially for nosocomial diseases, from Gram-positive staphylococci and streptococci to multidrug-resistant Gram-negative bacteria over the past decades is a direct result of the drying up of the antibiotic discovery pipeline (2). Broad-spectrum tetracyclines and aminoglycosides are commonly found among actinomycetes and were discovered by the 1950s. The last class of compounds acting against Gram-negative bacteria, the synthetic fluoroquinolones, was discovered in the 1960s. It is particularly difficult to discover antibiotics acting against Gram-negative species with their formidable penetration barrier (3). The network of charged lipopolysaccharides of the outer membrane restricts the penetration of large and hydrophobic compounds, and multidrug-resistant (MDR) pumps extrude molecules that leak through. The inner membrane is a barrier for hydrophilic compounds. As a result, few compounds manage to penetrate. Actinomycetes have been overmined for compounds acting against Gram-negative bacteria (2). Finding novel antibiotics proved to be extremely challenging (4, 5). A very large, continued effort to screen actinomycetes resulted in the discovery of narrow-spectrum daptomycin in the 1970s. Extensive screenings of millions of compounds in synthetic libraries have not produced viable leads (6, 7).

Despite obstacles and challenges, there have been several recent encouraging developments in antibiotic discovery. Analysis of penetration of chemically unrelated compounds into E. coli produced the first “rules of permeation” (8, 9). One simple rule is that charged amino groups favor diffusion through porins, and an aminated narrow-spectrum synthetic inhibitor of FabI (Debio-1452) is now in development against Gram-negative bacteria (8). Modification of narrow-spectrum arylomycin by adding positive charges resulted in a broad-spectrum compound (10). A simultaneous optimization of binding to target and penetration through porins led to the rational design of a novel broad-spectrum inhibitor of penicillin-binding proteins (11). Iboxamycin, a new protein synthesis inhibitor based on clindamycin, has excellent coverage of Gram-negative pathogens (5). Screening outside actinomycetes led to the discovery of teixobactin from an uncultured bacterium, *Eleftheria terra*, a novel member of Gram-negative betaproteobacteria (12). Teixobactin binds to lipid II and is the first systemically acting antibiotic without detectable resistance. The spectrum of this large compound is largely restricted to Gram-positive species.

One of the reasons actinomycetes are so successful in producing secondary metabolites is that a considerable part of their large genomes is devoted to biosynthetic gene clusters (BGC) (13). This is rare but not unique among bacteria. Deltaproteobacterial *Myxococcus* can harbor up to 46 BGCs (14), and gammaproteobacterial *Xenorhabdus* and *Photorhabdus*, for example, contain up to 41 BGCs (15). *Xenorhabdus* and *Photorhabdus*, symbionts of nematode microbiome, are of particular interest. They release antimicrobials when the nematode invades insect larvae (15, 16), and Gram-negative bacteria are the main competitors in that environment (17). We recently described darobactins, a novel class of antibiotics acting against Gram-negative bacteria produced by *Photorhabdus* (18, 19). Darobactins target BamA, the essential chaperone and insertase of outer membrane proteins.

In this study, we describe aminodeoxyguanosine (ADG), a novel antibiotic acting against E. coli that was discovered by a differential screen of *Photorhabdus* isolates. ADG is an interesting prodrug mimic of guanosine and is produced by a silent operon of Photorhabdus luminescens.

## RESULTS

### Isolation and identification of ADG.

*Xenorhabdus* and *Photorhabdus* were previously screened for antimicrobials, but the number of compounds identified is a small fraction of BGCs found in their genomes. To access silent operons, we have been screening concentrated extracts of these bacteria. Darobactin was discovered in such a screen from a 15× concentrated extract of *Photorhabdus khanii* (18). Apart from low production levels, another significant obstacle is the presence of nonspecific compounds produced by microorganisms. To focus on target-specific antimicrobials, we apply differential screening against two different bacterial species. Here, we screened *Xenorhabdus* and *Photorhabdus* extracts against E. coli and S. aureus. Selective action against E. coli leads to compounds that hit a specific target in Gram-negative bacteria.

A collection of 60 *Xenorhabdus* and *Photorhabdus* strains (see Table S1a in the supplemental material) was cultured in two different media, tryptic soy broth or Luria-Bertani (LB) broth, for 8 days. Supernatants were then concentrated 20× and spotted onto agar overlaid with either E. coli or S. aureus as a counterscreen. Several *Photorhabdus* isolates produced zones of inhibition with selective activity against E. coli that we determined to be caused by darobactin upon dereplication; however, we could not find any E. coli selective activity from a novel hit using this approach. Using much more concentrated extracts is impractical, since in our experience components of complex medium inhibit growth at high concentrations. Therefore, we subjected each 20× concentrated culture supernatant to high-performance liquid chromatography (HPLC) separation, producing 48 fractions for each isolate. Following HPLC fractionation, we dried and then solubilized the fractions in water so that the final concentration of each compound in a fraction would be ~200× that of the initial culture supernatant. This resulted in a total of 2,880 concentrated fractions.

10.1128/mbio.00700-22.7 TABLE S1 (a) ID and library source of the strains screened in this study. (b) E. coli enzymes that require GTP according to Brenda Enzyme Database. Download Table S1, XLSX file, 0.02 MB. Copyright © 2022 Shahsavari et al. 2022 Shahsavari et al. https://creativecommons.org/licenses/by/4.0/ This content is distributed under the terms of the Creative Commons Attribution 4.0 International license.

**FIG 1:**
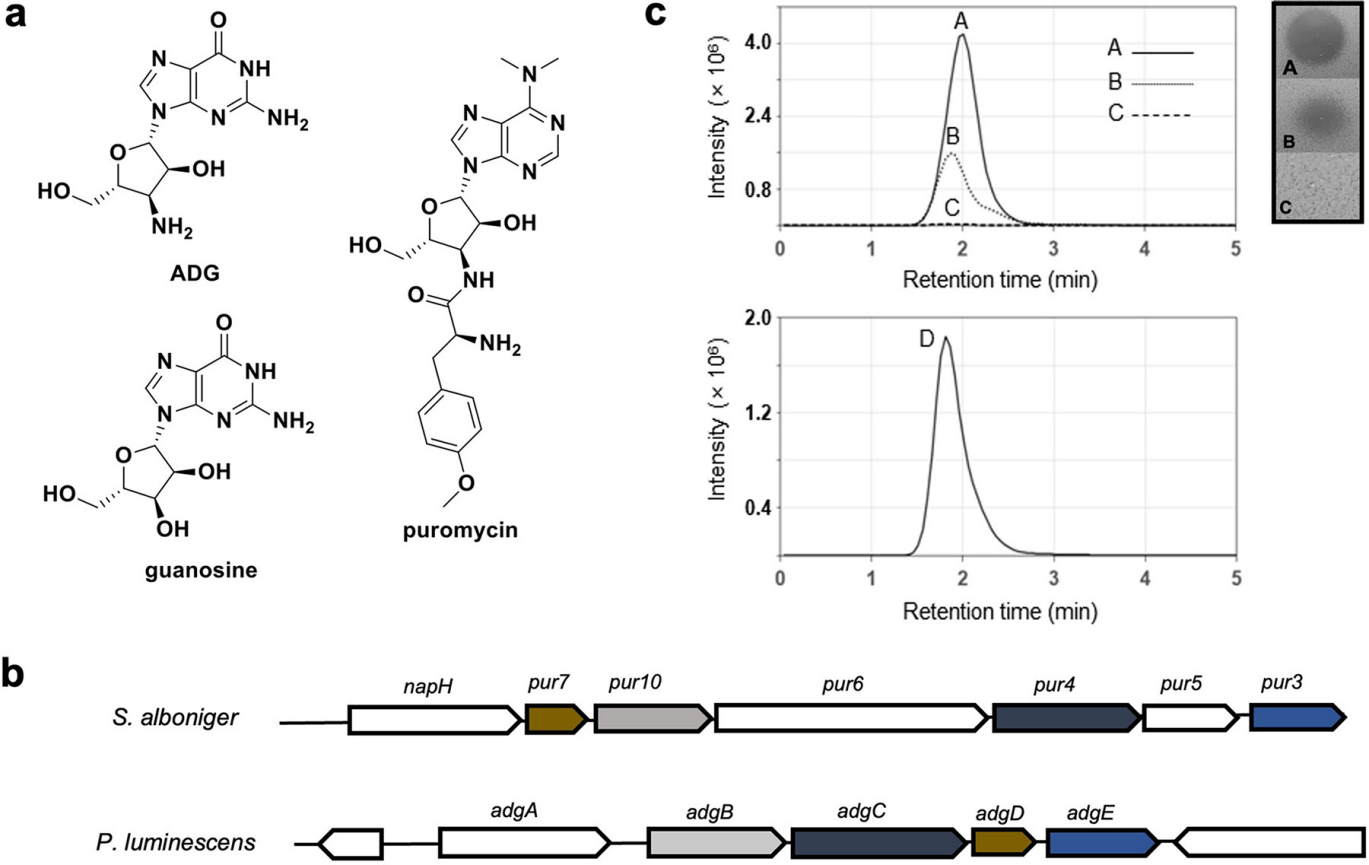
ADG structure and biosynthetic gene cluster (BGC). (a) Structures of ADG, guanosine, and puromycin. (b) Comparison between the puromycin BGC from *S. alboniger* and the ADG BGC from *P. luminescens*. The color-coded genes highlighted are homologs between the two BGCs. (c) Heterologous expression of ADG. Extracted ion chromatogram (EIC; *m/z* 283.11 to 283.19) on the left and inhibitory activity on E. coli lawn on the right of ADG standard (60 ng/mL ADG in H_2_O) (line A), partially purified extract of E. coli Bap1+pNS-ADG (line B), partially purified extract of E. coli Bap1+pRSFduett-1 (negative control) (line C), and EIC (*m/z* 283.11 to 283.19) of coinjection of lines A and B (line D).

Three adjacent HPLC fractions of *P. luminescens* KLE11358 produced a zone of inhibition on an E. coli lawn, while there was no inhibition of S. aureus, and we decided to follow up on this hit. Bioassay-guided isolation of the extract using several HPLC conditions (described in detail in Materials and Methods) led to the purification of an active fraction (Fig. S1a). Mass spectrometry (MS) analysis showed that it contains a compound with a mass of [M+H]^+^ = 283.11 (Fig. S1b). Mass spectrometry fragmentation and nuclear magnetic resonance (NMR) studies led to the identification of the active compound, which is 3′-amino-3′-deoxyguanosine (ADG) (Fig. 1a and Fig. S2). ADG is a modified guanosine in which the 3′ hydroxyl is replaced by an amino group. Modified nucleosides have long been of interest in the field of antiviral therapeutics (20), and a variety of nucleoside analogs, including ADG, have been synthesized for this purpose (21). We confirmed that the synthetic form of the compound has an identical mass spectrometry and NMR profile and biological activity, and we decided to use it for further experiments (Table S2 and Fig. S1b and S3).

10.1128/mbio.00700-22.1 FIG S1 (a) HPLC chromatogram and activity of pure ADG that causes a zone of inhibition on E. coli lawn with no activity on S. aureus lawn in the activity lawn assay. (b) HRESIMS spectra of (A) pure ADG and (B) authentic synthetic ADG. Download FIG S1, DOCX file, 0.8 MB. Copyright © 2022 Shahsavari et al. 2022 Shahsavari et al. https://creativecommons.org/licenses/by/4.0/ This content is distributed under the terms of the Creative Commons Attribution 4.0 International license.

10.1128/mbio.00700-22.2 FIG S2 (a) ^1^H-NMR spectrum of ADG in D_2_O (400 MHz). (b) ^1^H-^1^H COSY spectrum of ADG in D_2_O (400 MHz). (c) ^13^C-NMR spectrum of ADG in DMSO-*d*_6_ (100 MHz). (d) ^1^H-NMR chemical shifts of ADG and ^1^H-^1^H COSY correlations (D_2_O, 400 MHz). Download FIG S2, DOCX file, 0.9 MB. Copyright © 2022 Shahsavari et al. 2022 Shahsavari et al. https://creativecommons.org/licenses/by/4.0/ This content is distributed under the terms of the Creative Commons Attribution 4.0 International license.

10.1128/mbio.00700-22.3 FIG S3 (a) ^1^H-NMR (400 MHz) in D2O of (A) natural product ADG, (B) synthetic ADG, and (C) 1:1 ratio mixture of natural and synthetic ADG. The left shows the spectra from0 to 10 ppm, the right shows the spectra from 3 to 4.5 ppm. (b) UV spectra of natural (left) and synthetic ADG (right). Download FIG S3, DOCX file, 0.3 MB. Copyright © 2022 Shahsavari et al. 2022 Shahsavari et al. https://creativecommons.org/licenses/by/4.0/ This content is distributed under the terms of the Creative Commons Attribution 4.0 International license.

### Identification of biosynthetic gene cluster and heterologous expression.

BGCs usually build a product sequentially starting from a simple precursor. ADG, however, differs from guanosine by a single small substitution, and identifying its BGC is not obvious. Fortunately, there is a well-studied antibiotic, puromycin, that is a heavily modified nucleoside, and we reasoned that its operon may share some homology with the BGC of ADG. Specifically, ADG and puromycin share a 3′-aminodeoxyribose moiety (Fig. 1a). Hence, the puromycin BGC (GenBank accession no. X92429.1) was subjected to BLAST search against the *P. luminescens* genome, reporting a 5-gene putative BGC. These genes were termed *adgA-E*, with *adgB-E* being homologous to *pur10*, *pur4*, *pur7*, and *pur3* respectively, and *adgA* was annotated as an MFS transporter gene (Fig. 1b and Table S3). Due to the low GC content of the BGC in contrast to the adjacent genes, its borders were easily defined, and we hypothesized that AdgB-E converted GTP into ADG in 3 to 4 biosynthetic steps (Fig. 1b), while the MFS transporter AdgA exported ADG out of the cell. The biosynthetic steps are proposed based on the reported pathway of puromycin biosynthesis in Streptomyces alboniger (22). To biologically synthesize ADG, the precursor GTP is first oxidized by AdgB at the 3′ position, followed by dephosphorylation by AdgD to remove two phosphate groups. AdgC facilitates the amination at the 3′ position before AdgE removes the remaining phosphate group (Fig. S4). To test this assumption, we heterologously expressed this BGC in E. coli. We constructed a plasmid containing the biosynthetic portion of the operon (*adgB-E*) under the control of an isopropyl-β-d-thiogalactopyranoside (IPTG)-inducible promoter (pNS-ADG). Following confirmation of the desired plasmid construct, we transformed E. coli BAP1 with this plasmid. We also transformed E. coli with the empty PRSF vector as a negative control (E. coli Bap1+pRSFduett-1). Expression of the *adgB-E* operon was induced by IPTG, and upon LC-MS analysis, a compound with a mass matching ADG and with the same retention time as the ADG standard was identified in the culture supernatant of E. coli BAP1+pNS-ADG (Fig. 1c). This compound was absent from the strain with an empty vector. This result confirms *adgB-E* as the biosynthetic gene cluster for the production of ADG. This operon apparently remains fairly silent in E. coli, and the low level of ADG production does not cause toxicity.

10.1128/mbio.00700-22.4 FIG S4 Proposed biosynthetic pathway for ADG in *P. luminescence.* In the first step, AdgB oxidizes the precursor guanosine triphosphate at the 3’ position. In the second step, AdgD removes two phosphate groups. In the third step, AdgC facilitates amination at the 3’ position. In the last step, AdgE removes the remaining phosphate group. Download FIG S4, DOCX file, 0.1 MB. Copyright © 2022 Shahsavari et al. 2022 Shahsavari et al. https://creativecommons.org/licenses/by/4.0/ This content is distributed under the terms of the Creative Commons Attribution 4.0 International license.

10.1128/mbio.00700-22.9 TABLE S3 Biosynthetic enzymes of ADG compared to puromycin BGC from *S. alboniger*. Download Table S3, DOCX file, 0.10 MB. Copyright © 2022 Shahsavari et al. 2022 Shahsavari et al. https://creativecommons.org/licenses/by/4.0/ This content is distributed under the terms of the Creative Commons Attribution 4.0 International license.

### Biological activity and toxicity.

ADG showed a MIC of 4 μg/mL against E. coli ATCC 2592, a clinical isolate. The lab strain E. coli MG1655 showed a higher MIC of 16 μg/mL, while E. coli W0153, which is defective in lipopolysaccharide production and has a knockout in TolC, had a MIC of 4 μg/mL. This compound also showed moderate activity against Klebsiella pneumoniae (MIC, 16 μg/mL). There was little activity against gut bacteria and Gram-positive S. aureus (Table 1). These findings confirm the results of differential screening and show that ADG acts selectively against Gram-negative bacteria. We checked the 50% inhibitory concentration (IC_50_) of ADG against mammalian cell lines FaDu and HepG2, and ADG started to show cytotoxicity at 128 μg/mL.

**TABLE 1:** MICs for ADG*^a^*

Organism and genotype	Concentration (μg mL^−1^)	Presence (+) or absence (−) of bacterial Gsk
Pathogenic bacteria (MIC)		
*Escherichia coli* ATCC 25922	4	+
*Escherichia coli* W0153	4	+
*Escherichia coli* MG1655	16	+
*Escherichia coli* AR350 (*mcr-1*)	64	+
*Klebsiella pneumoniae* ATCC 700603	16	+
*Klebsiella pneumoniae* ESBL JMI 1052654	16	+
*Pseudomonas aeruginosa* PAO1	>128	−
*Acinetobacter baumannii* ATCC 17978	>128	−
*Staphylococcus aureus* HG003	>128	−
*Escherichia coli* BW25113	32	+
*Escherichia coli* BW25113 Δ*gsk*	>128	−
*Escherichia coli* AG1 pCA24N-gsK without IPTG	32	+
*Escherichia coli* AG1 pCA24N-gsK with 0.1 mM IPTG	4	+
*Escherichia coli* MG1655 *gsk*^+^^+^	2	+
Gut bacteria		
*Klebsiella variicola* KLE 2552*^b^*	32	+
*Veillonella ratti* KLE 2365*^b^*	>128	−
*Clostridium bifermentans* KLE 2329*^b^*	>128	−
*Clostridium hylemonae* KLE 2503*^b^*	>128	−
*Enterococcus faecalis* KLE 2341*^b^*	>128	−
*Stenotrophomonas maltophilia* KLE 11416*^b^*	>128	−
Human cell lines (IC_50_)		
FaDu	128	NA
HepG2	128	NA

a E. coli W0153, AB1157 *asmB1* Δ*tolC*::*kan mcr-1*, polymyxin resistant. ESBL, extended-spectrum β-lactamase. JMI, JMI Laboratories. NA, not applicable.

b Cultivated under anaerobic conditions. Human stool isolate, K. Lewis laboratory collection.

### ADG is a prodrug.

**FIG 2:**
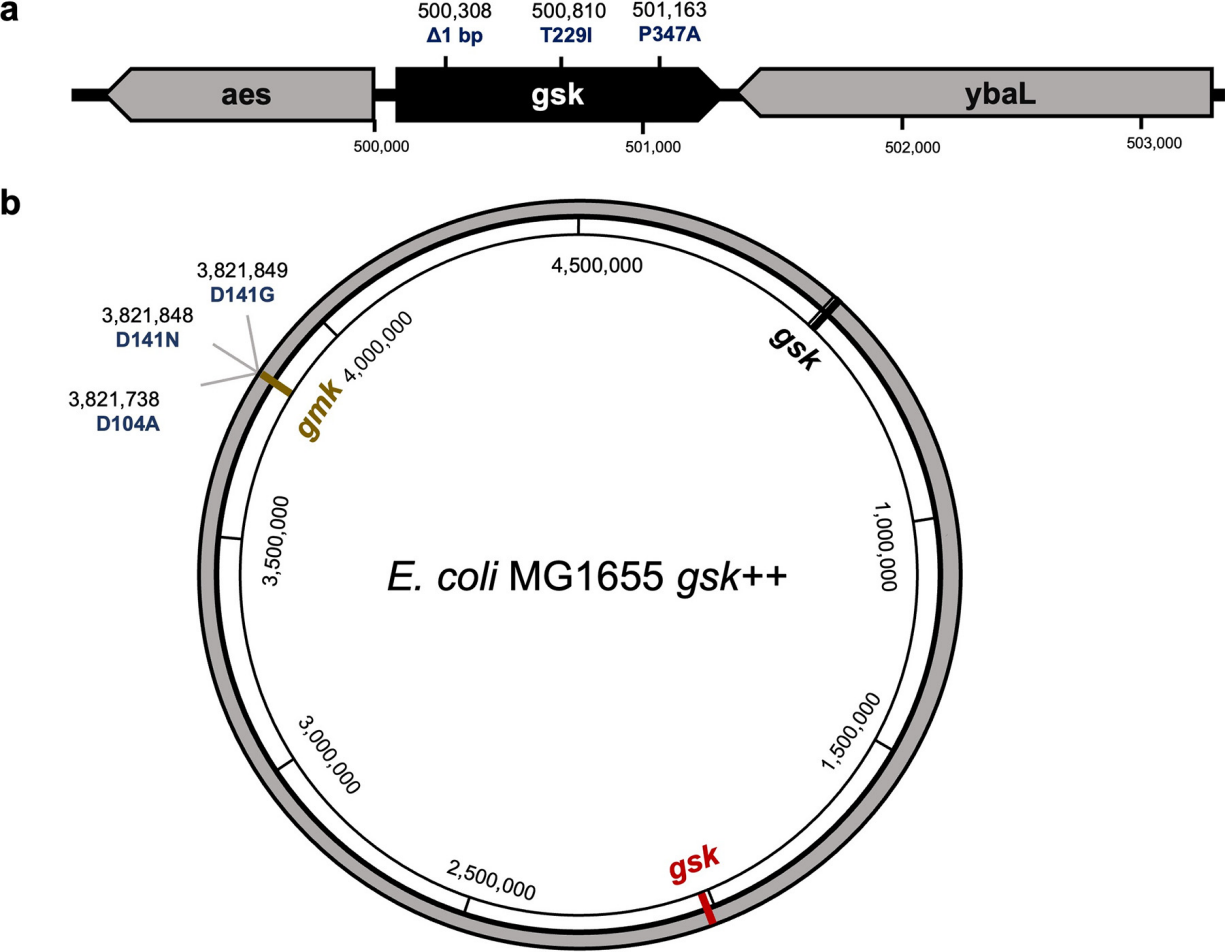
Chromosomal mutations that confer resistance to ADG in E. coli. (a) Mutations in *gsk* conferring resistance to ADG. (b) Mutations in E. coli MG1655 *gsk++* conferring resistance to ADG.

To identify the target of ADG, we selected for resistant mutants by plating 1.8 × 10^6^ E. coli cells on nutrient agar medium containing ADG at 4× MIC. The frequency of resistance was 6.5 × 10^−5^, which is fairly high for an antibiotic, indicative of a null mutation (23). We picked three colonies and measured their ADG MIC. All three mutants were highly resistant, with a MIC of >128 μg/mL. Whole-genome sequencing with Illumina showed mutations in the *gsk* gene for all isolates (Fig. 2a). In one of the resistant mutants (RM1) there was a single frameshift base pair deletion in the N-terminal region of the open reading frame (ORF). Two other resistant mutants, RM2 and RM3, showed base substitutions T229I and P347A, respectively. Gsk is a guanosine/inosine kinase and the first enzyme of a purine salvage pathway that produces nucleoside triphosphates. It uses ATP to phosphorylate guanosine or inosine, producing GMP or IMP, respectively. An E. coli BW25113 *Δgsk* mutant from the Keio collection was fully resistant to ADG. Next, we determined the MIC of E. coli AG1 pCA24N*-gsK* from the ASKA overexpression library, which contains a high-copy-number plasmid carrying *gsk*. Induction of *gsk* expression with IPTG resulted in an 8-fold increase in susceptibility to ADG (Table 1). These results suggested that ADG is a prodrug that is activated by Gsk. It is important to note that Gsk is not essential and guanosine can be synthesized *de novo* in E. coli (24), allowing for the formation of null mutants. The enzyme is also specific to E. coli and its close relatives, explaining the selectivity of ADG.

**FIG 3:**
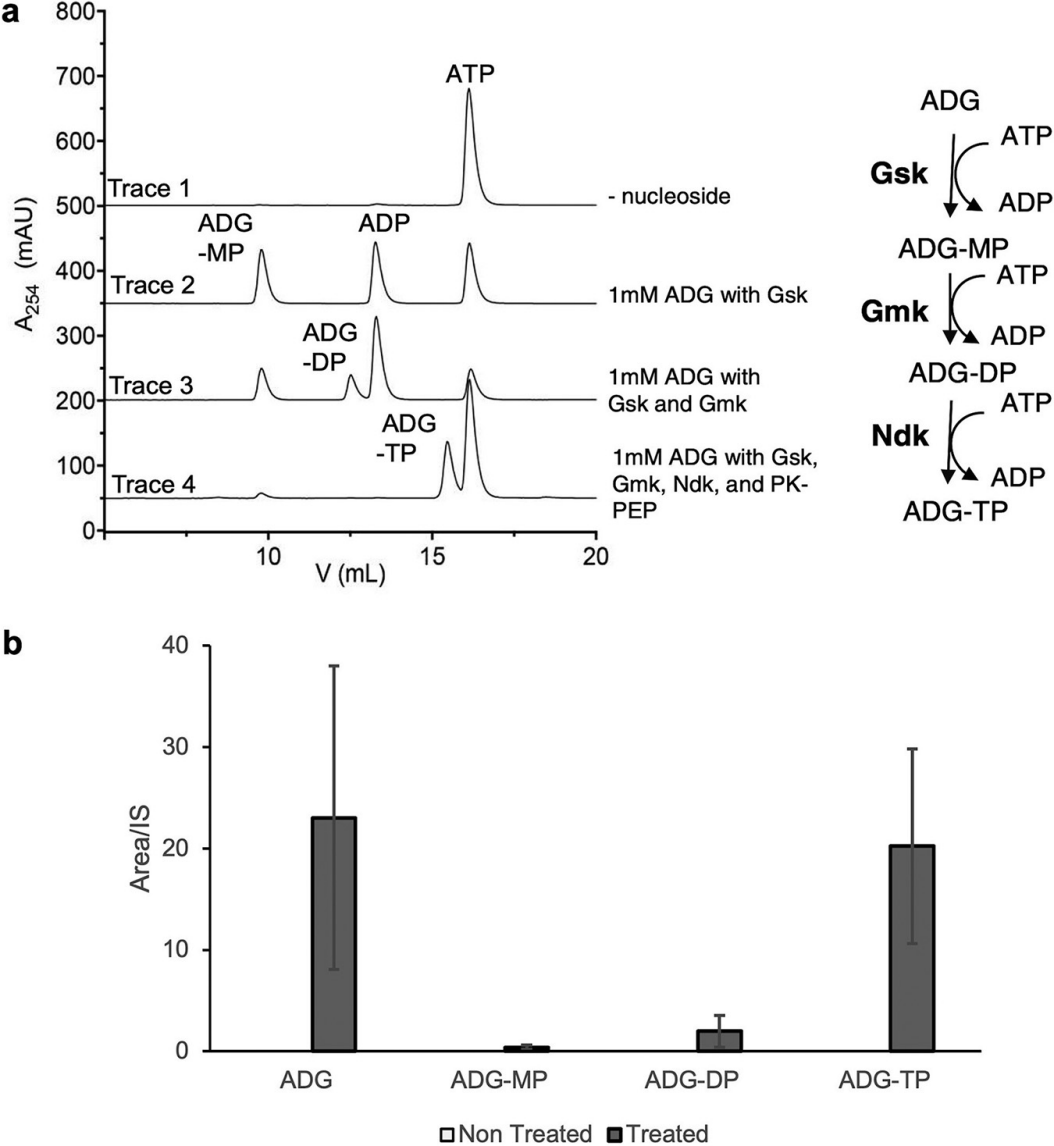
Phosphorylation of ADG *in vitro* and *in vivo.* (a) Consecutive phosphorylation of ADG. (Left) *In vitro* phosphorylation of ADG using recombinant Gsk, Gmk, and Ndk. (Right) Schematic cascade of phosphorylation of ADG. Gsk, guanosine/inosine kinase; Gmk, GMP kinase; Ndk, nucleoside diphosphate kinase; PK, pyruvate kinase; PEP, phosphoenolpyruvate; ADG-MP, ADG-DP, and ADG-TP, ADG monophosphate, diphosphate, and triphosphate, respectively. (b) Targeted mass spectrometry analysis of E. coli metabolome extracts. Presence of ADG-MP, ADG-DP, and ADG-TP mass candidates in cell extract metabolome 1 h after treatment with ADG. Data are means from three biological replicates. Error bars are standard deviations from the means. The mass of ADG and its nucleotide derivatives were not detected in the nontreated samples.

Since ADG has a structure similar to that of guanosine, we reasoned that Gsk can phosphorylate ADG into ADG-5′-monophosphate (ADG-MP) (Fig. 3a, right). To perform an *in vitro* phosphorylation assay, we cloned the *gsk* open reading frame (ORF) into pET Sumo vector downstream of a His_6_-Sumo ORF and used E. coli BL21(DE3) for expression. Following purification of Gsk and removal of the His_6_-Sumo tag, we used this enzyme to phosphorylate ADG. Indeed, when treated with ATP and purified with recombinant Gsk, ADG is converted to ADG-MP *in vitro*, with concomitant production of ADP (Fig. 3a, compare traces 1 and 2). At a concentration of 1 mM, ADG phosphorylation is about 40-fold slower than that of guanosine, the preferred substrate of Gsk (Fig. S5). Despite the slow kinetics, this result suggested that ADG is similar enough to guanosine to be recognized by this promiscuous enzyme. We then considered that other E. coli enzymes may further phosphorylate ADG-MP into 5′-diphosphate (ADG-DP) and 5′-triphoshate (ADG-TP) by E. coli guanylate kinase (Gmk) and nucleoside diphosphate kinase (Ndk), respectively (Fig. 3a, right). To test this, again we used the pET28b vector and E. coli BL21(DE3) to clone and express Gmk and Ndk and then purified them using the nickel nitrilotriacetic acid (Ni-NTA) affinity procedure. Remarkably, inclusion of the purified Gmk and Ndk in addition to Gsk in an *in vitro* assay produced ADG-DP and ADG-TP (Fig. 3a, traces 3 and 4). Furthermore, in a metabolomic analysis, we investigated E. coli metabolome extracts after treatment with ADG for its nucleotide’s derivatives. We collected the metabolome extracts before and 1 h after treatment with ADG, subjected them to LC-MS analysis, and looked for mass of ADG and mass of candidates of ADG nucleotides. The results clearly revealed the production of ADG-MP, ADG-DP, and ADG-TP *in vivo* in the presence of ADG (Fig. 3b). These data suggest that ADG acts as a prodrug: it requires endogenous E. coli enzymes to convert it into an active compound, ADG-TP.

10.1128/mbio.00700-22.5 FIG S5 (a) Kinetics traces of ADG phosphorylation by Gsk compared to guanosine (Guo). Production of ADP is coupled to consumption of NADH and is monitored as the decrease of absorbance at 340 nm. (b) Initial reaction velocity. Error bars are SD from three reaction mixtures set up in separate wells on the same 96-well plate. (c) Identity of AAG, AA(ADG), and AAGA confirmed using mass spectrometry. Download FIG S5, DOCX file, 0.2 MB. Copyright © 2022 Shahsavari et al. 2022 Shahsavari et al. https://creativecommons.org/licenses/by/4.0/ This content is distributed under the terms of the Creative Commons Attribution 4.0 International license.

### Identification of the mechanism of action.

The resistant mutants we identified pointed to the unexpected nature of ADG mode of action but did not lead to the target. Finding rare mutations in an essential target would be very difficult given the large background of null mutants in *gsk* that exhibit full resistance to ADG. We therefore used the E. coli AG1 Gsk overexpression strain from the ASKA library to increase the probability of finding target mutations. We tried to select for resistant mutants by plating E. coli cells carrying additional copies of *gsk* on medium with ADG at 4× MIC. However, there was no change in the MIC of the colonies following passage to fresh medium, suggesting that the plasmids are not stable. To obtain a stable E. coli strain with two copies of *gsk*, we introduced a second copy into the neutral *fliT* gene region (25) of the E. coli chromosome using λ Red recombination (26). We plated 1.5 × 10^9^ E. coli *gsk*++ cells on medium with a high concentration of ADG, 64× MIC. The frequency of resistance to ADG in this strain was fairly low, 8.2 × 10^−9^, but very close to the theoretical frequency of mutating both copies of *gsk* (6.5 × 10^−5^ × 6.5 × 10^−5^ = 4.225 × 10^−9^). Intriguingly, however, all three resistant mutations mapped to *gmk*, which encodes guanylate kinase, the second enzyme that adds phosphate to ADG-MP (Fig. 2b and Fig. 3a, right). Unlike Gsk, Gmk is essential (27), and that is why we did not obtain null mutants in *gmk.* These Gmk mutant proteins likely no longer bind ADG-MP. We did not find resistant mutants in Ndk that make the final product, ADG-TP. Apparently, mutations that would discriminate between ADG-DP and GDP are very rare or nonexistent. Finding resistant mutants in Gmk supports the idea of ADG being a prodrug, but this experiment did not identify its target.

**FIG 4:**
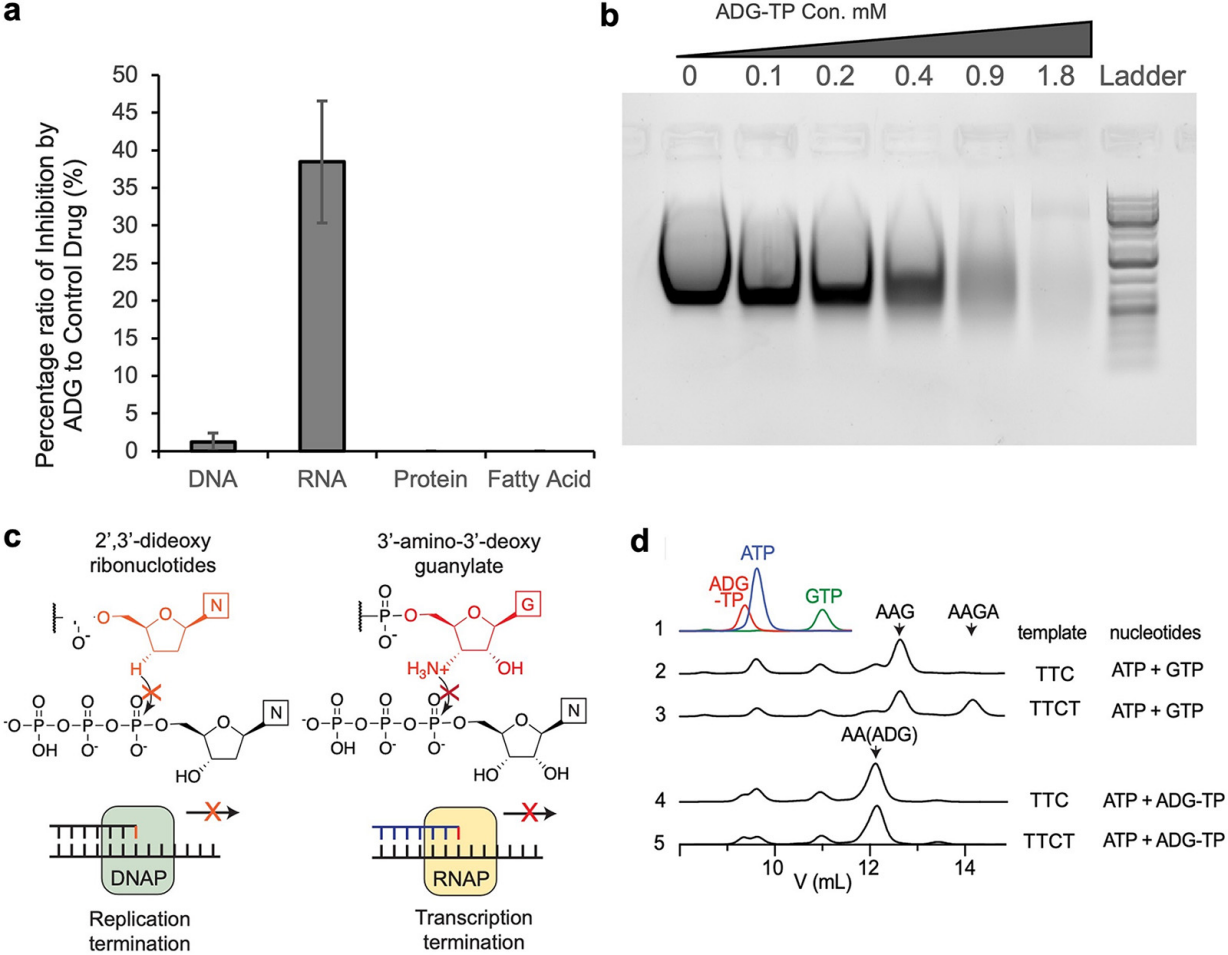
Elucidation of ADG mode of action. (a) Impact of ADG on macromolecular biosynthesis of E. coli. Incorporation of [^14^C]thymidine (DNA), [^14^C]uridine (RNA), [^14^C]l-amino acid mixture (protein), and [^14^C]acetic acid (fatty acid) was determined in cells treated with ADG at 2× MIC. Ciprofloxacin (2× MIC), rifampicin (2× MIC), chloramphenicol (2× MIC), and triclosan (2× MIC) were used as controls. Means from three biological replicates are shown. Error bars are standard deviations from the means. (b) *In vitro* inhibition of RNA synthesis by ADG-TP. The effect of 2-fold dilutions of ADG-TP in an *in vitro* RNA synthesis reaction mixture was evaluated by agarose gel electrophoresis. This experiment was repeated three times. This gel is a representative of three experiments. (c) Diagram showing the similarity between termination of DNA synthesis by ddNTP (left) and termination of RNA synthesis by ADG-TP (right). (d) Anion-exchange traces of *in vitro* transcription reactions. Standard traces for starting-material nucleotides are included on the top. Peak positions of major oligonucleotide products (with 5′-triphosphate) are indicated by arrows. Identity of AAG, AA(ADG), and AAGA were confirmed using mass spectrometry (Fig. S5c).

A general approach to determine the mode of action of a new antimicrobial is to follow its effect on label incorporation into major biosynthetic pathways. Addition of ADG to E. coli led to a considerable inhibition of RNA synthesis (Fig. 4a). Other major pathways, including the synthesis of proteins or fatty acids, were largely unaffected (Fig. 4a). We then directly examined the activity of ADG-TP on transcription *in vitro*. We generated ADG-TP and added it to the *in vitro* transcription reaction mixtures containing a DNA template, RNA polymerase, and ribonucleotides in a 2-fold dilution manner. As the concentration of ADG-TP increases in the reaction, less RNA was synthesized, as shown by agarose gel electrophoresis (Fig. 4b).

The impact of ADG-TP on transcription is reminiscent of the 2′,3′-dideoxyribonucleotides used for Sanger sequencing, which terminates elongation by DNA polymerase due to the lack of the 3′-hydroxyl group (Fig. 4c). Since the 3′-amino group in ADG remains nucleophilic when not protonated, we performed additional *in vitro* transcription assays to test if elongation upon a 3′-amino group is possible. When using a template with the sequence **TTC**GGAGCGAG and adding only ATP and GTP to the reaction mixture, RNA polymerase efficiently generates the trinucleotide, AAG, before termination due to the lack of CTP in the reaction mix (Fig. 4d, trace 2). Under the same condition, a second template with the sequence **TTCT**GGAGCGAG gave rise to the tetramer, AAGA, as expected, although AAG, now an abortive transcription product, was produced at an even larger amount than AAGA (Fig. 4d, trace 3), likely as a result of the well-characterized, high abortive initiation rate of T7 RNA polymerase (28). Remarkably, while ADG-TP was efficiently incorporated into the trimer AA(ADG) (Fig. 4d, trace 4), no tetrameric AA(ADG)A was observed when transcribing the second template (Fig. 4d, trace 5), showing that it is impossible to elongate transcription beyond a 3′-ADG nucleotide. Thus, we envision that incorporation of ADG nucleotide into RNA *in vivo* should lead to widespread abortive transcription and severe reduction of full-length, functional transcripts, which could be far more detrimental to fitness than inhibition of RNA polymerase.

**FIG 5:**
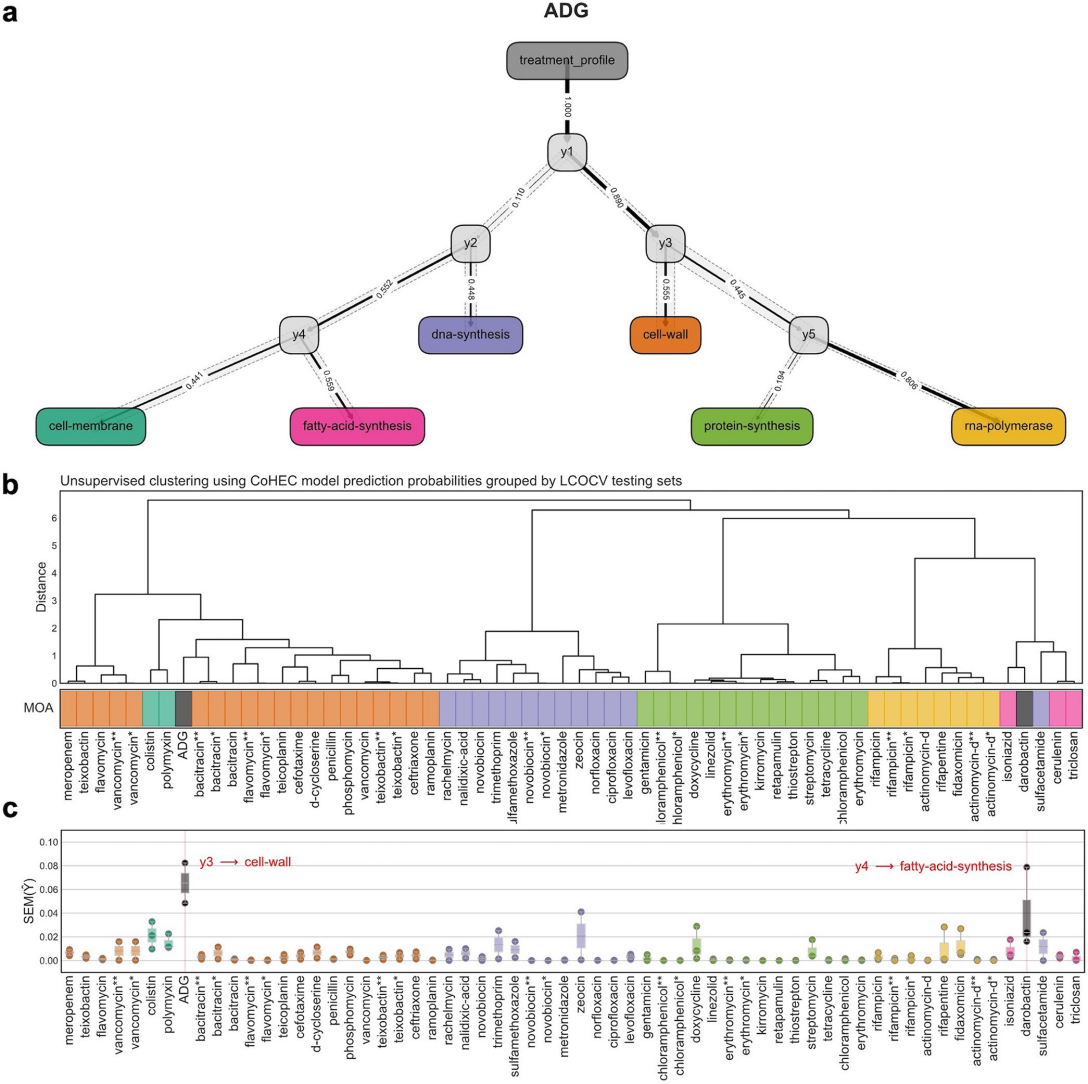
Predictive modeling based on compounds with known modes of action. (a) CoHEC model decision graph for ADG representing MOA predictions. Prediction paths where each terminal colored node depicts an MOA, each internal gray node represents a submodel decision point, the solid line edge width corresponds to the probability according to the model for the respective path, and the dotted opaque path represents standard errors along the decision path. (b) Unsupervised hierarchical clustering of held-out test set prediction probabilities for each compound unobserved by the model. (c) Standard error profiles for each of the submodel predictions for the held-out test compounds. ADG and darobactin are flagged with novel activity by their high standard error profiles and uncertainty with respect to the model.

While ADG-TP targets transcription, inhibition of RNA synthesis is incomplete, about 40% compared to that of rifampicin. This incomplete inhibition is likely due to an insufficient concentration of ADG-TP in the cytoplasm to completely inhibit RNA synthesis. At the same time, ADG is fairly potent, with a MIC of 2 μg/mL against an E. coli strain overexpressing Gsk. Notably, we did not find resistant mutations in the RNA polymerase. One intriguing possibility is that ADG-TP hits multiple targets, and this accounts for its potency and the inability to obtain target mutations. ADG-TP mimics GTP, and there are at least 80 enzymes in E. coli that require GTP either as a substrate, cofactor, activator, or inhibitor according to the Brenda Enzyme Database (Table S1b). It is clear that ADG-TP does not interfere with all of these processes; for example, it had no detectable effect on protein synthesis, which requires GTP for translation. In line with this, transcriptome analysis and predictive modeling based on known mode of action (MOA) showed no confident inhibition of a single pathway (Fig. 5a). More specifically, predictive models designed to classify the primary MOA based on transcriptomics in a stepwise manner suggest that the compound hits one or more targets that are not defined within the classical MOA categories based on known antibiotics. The predictive model output is a decision graph with variance profiles around each decision in the prediction. The prediction based on this model revealed high uncertainty along the decision path with exceptionally high variance and low probability at the terminal prediction. This model has been used previously to flag the novelty of darobactin (29), which exhibits a variance profile similar to that of ADG, further suggesting either a single new target or multiple targets (Fig. 5).

**FIG 6:**
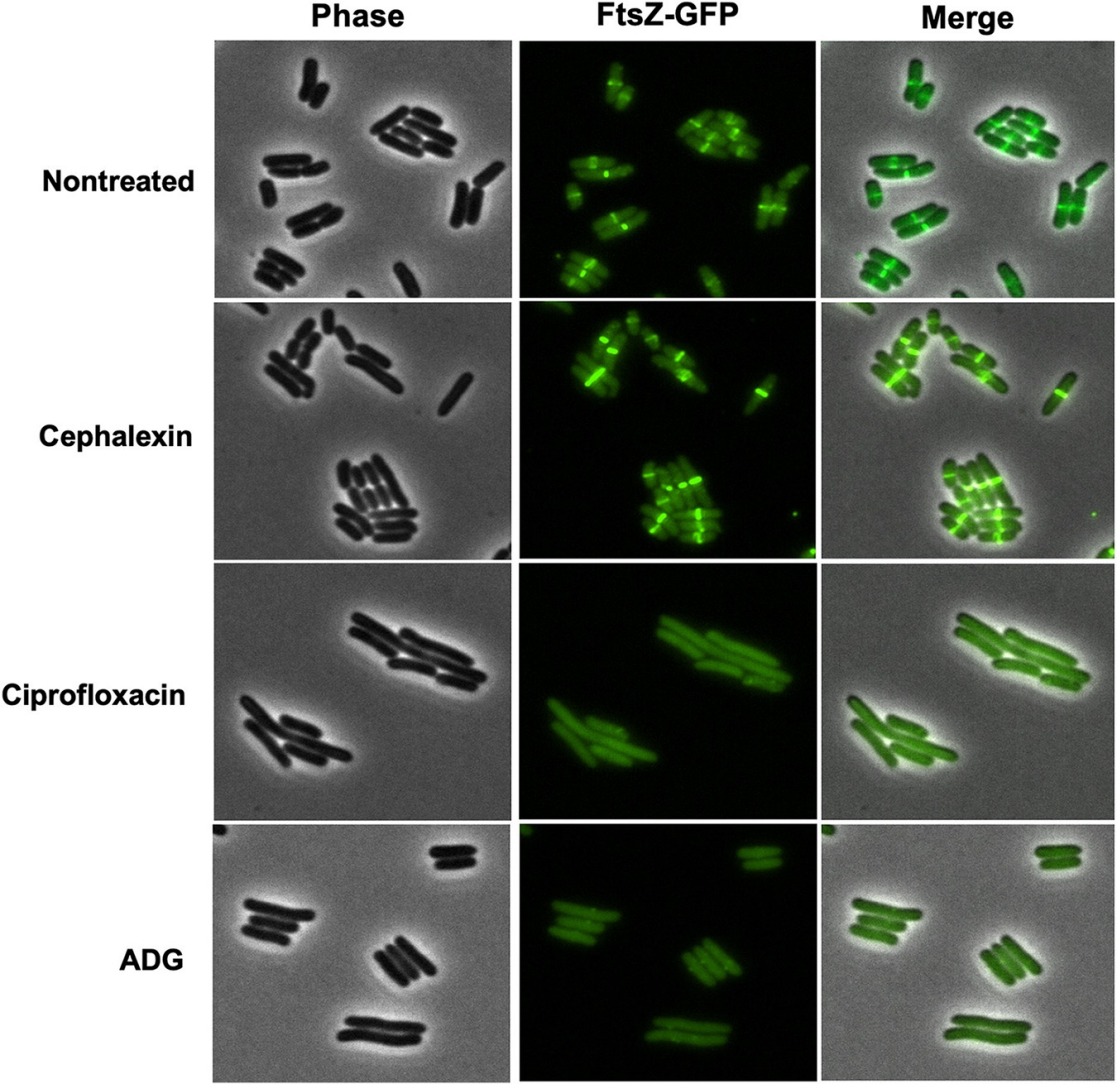
FtsZ ring localization and cell division is inhibited by ADG. ADG and ciprofloxacin inhibit localization of FtsZ at the division site. E. coli MG1655 FtsZ-GFP was grown and treated with either ADG (128 μg/mL), ciprofloxacin (1 μg/mL), cephalexin (8 μg/mL), or no drug for 1 h at 37°C. Cells were spotted onto a 1.5% low-melting-point agarose pad and observed with a fluorescence microscope. This experiment was repeated three times. This image is a representative of three independent experiments.

Keeping in mind the possible multitargeting by ADG, we noticed that E. coli cells treated with ADG tend to elongate (Fig. S6, Video S1), which suggested that cell division was impaired. The major cell division protein, FtsZ, is a tubulin homolog that polymerizes into a ring-like structure (Z-ring) (30). Contraction of the Z-ring allows the separation of the two daughter cells. Similarly to tubulin, FtsZ has GTPase activity, with GTP hydrolysis powering its polymerization. We then used a translational fluorescent fusion of FtsZ (FtsZ-GFP) to assess the localization of FtsZ and the assembly of the Z-ring in the presence of ADG. In control cells, FtsZ formed a characteristic band in the middle of the cells (Fig. 6). We used ciprofloxacin as a positive control, as this antibiotic inhibits DNA gyrase (31), which consequently induces the SOS response, resulting in the expression of the cell division inhibitor SulA (32). SulA inhibits septation by blocking FtsZ polymerization and, thus, the assembly of the Z-ring (33, 34). Thus, in the presence of ciprofloxacin, FtsZ does not localize in a ring and cells elongate without division (Fig. 6). Cephalexin, used as a negative control, inhibits the transpeptidase FtsI and impedes the constriction of the Z-ring, which is trapped at mid-cell (35). In the presence of cephalexin, the Z-ring is clearly visible (Fig. 6). In the presence of ADG, FtsZ was delocalized, suggesting inhibition of polymerization (Fig. 6). Additional experiments will be required to establish a direct interaction between ADG-TP and FtsZ.

10.1128/mbio.00700-22.6 FIG S6 E. coli MG1655 cells were spotted onto a 1.5% agarose pad containing ADG (8× MIC), the membrane stain FM4-64 (false colored in magenta), and membrane permeabilization stain Sytox green (false-colored in green). Cells were incubated at 37°C in a thermostatic chamber and imaged every 15 min under the microscope. The selected time points and phase contrast images were chosen to best represent the elongation of E. coli MG1655 with ADG. Scale bars, 5 μm. This experiment was repeated three times. This image is representative of the three experiments. Download FIG S6, DOCX file, 0.3 MB. Copyright © 2022 Shahsavari et al. 2022 Shahsavari et al. https://creativecommons.org/licenses/by/4.0/ This content is distributed under the terms of the Creative Commons Attribution 4.0 International license.

10.1128/mbio.00700-22.10 VIDEO S1 E. coli MG1655 cells were spotted onto a 1.5% agarose pad containing ADG (8× MIC), the membrane stain FM4-64 (false colored in magenta), and membrane permeabilization stain Sytox green (false colored in green). Cells were incubated at 37°C in a thermostatic chamber and imaged every 15 min under the microscope. Phase-contrast images were chosen to best visualize the elongation phenotype of E. coli under ADG treatment. Scale bars, 5 μm. Download Movie S1, MOV file, 5.0 MB. Copyright © 2022 Shahsavari et al. 2022 Shahsavari et al. https://creativecommons.org/licenses/by/4.0/ This content is distributed under the terms of the Creative Commons Attribution 4.0 International license.

## DISCUSSION

Antibiotic discovery is uniquely challenging due to several factors: penetration barrier, especially in Gram-negative bacteria with their additional outer membrane; rapid acquisition of resistance to most compounds; and a very large background of nonspecifically acting compounds in both natural and synthetic collections (2). In the case of natural products, we have overmined actinomycetes, from which the main classes of compounds acting against Gram-negative bacteria were discovered. Current efforts are aimed at expanding the search outside actinomycetes and turning on silent operons.

In this study, we combine several approaches, i.e., a focus on an attractive group of producers, access to silent operons, and differential screening, to discover a novel prodrug antibiotic, aminodeoxyguanosine (ADG), that is converted into ADG-TP, a mimic of GTP. In search of compounds acting against Gram-negative bacteria, we focused on *Xenorhabdus* and *Photorhabdus*, symbionts of entomopathogenic nematode gut microbiomes (36). Several dozen antimicrobials have been identified over the past decades from these bacteria, and similar to other groups, the majority of these compounds are nonspecific (37). However, target-specific compounds have also been identified recently: odilorhabdins, novel inhibitors of translation (38); darobactins, inhibitors of BamA (18), the essential protein of the outer membrane; dynobactins, another class of BamA inhibitors (unpublished data); and the prodrug ADG of this study. Notably, all these compounds come from screening very small libraries, typically dozens of isolates, by comparison to 10^4^ to 10^6^ that have led to the discovery of the last useful/target-specific compounds to come out of industrial screens focused on actinomycetes, i.e., erythromycin, daptomycin, and platensimycin (39, 40). Another important distinction is that erythromycin, daptomycin, and platensimycin only act against Gram-positive bacteria, suggesting that actinomycetes have been exhaustively mined for compounds acting against Gram-negative bacteria, while odilorhabdins, darobactins, dynobactins, and ADG all act against Gram-negative species (19). This is probably not accidental, as the main competitors of *Xenorhabdus* and *Photorhabdus* are likely Gram-negative bacteria. The nematode gut is not anaerobic due to its small dimensions, and its main inhabitants are proteobacteria such as E. coli, Pseudomonas, and *Stenotrophomonas* (41). When the nematodes infect insect larvae, they release *Xenorhabdus* and *Photorhabdus* that produce antimicrobials to protect this food source, and the other Gram-negative gut bacteria become their immediate competitors.

The relative success in finding compounds acting against Gram-negative bacteria is limited by both the background of nonspecific compounds and by low production from the operons of *Xenorhabdus* and *Photorhabdus*, the majority of which are silent under laboratory conditions. The standard approach is to engineer the BGC, either in the producing organism or heterologously, for increased production (42). This becomes a separate project for each BGC and is very low throughput. To our knowledge, a novel target-specific compound has not yet emerged from this approach. We tested a simpler approach, screening concentrated extracts, and this led to identification of darobactin and dynobactin from their silent operons (43). This is much faster than engineering production, but there is a limit to how much an extract can be concentrated, typically 20× in our hands, before inhibition from medium components begins to interfere with the assay. Another problem is concentration of nonspecific compounds that will mask target-specific antibiotics. The majority of silent operons probably remain inaccessible to this approach.

In this project, we expanded screening of concentrated extracts to achieve a much higher concentration, avoid interference with nuisance compounds, and identify target-specific molecules in a focused manner. For this, 20× concentrated extracts from a small set of 60 *Xenorhabdus* and *Photorhabdus* isolates were separated into 48 fractions by HPLC, concentrated, and tested for activity by agar overlay. This achieves approximately 200× concentration, and separation into fractions resolves interference both by medium components and by nonspecific compounds. To identify target-specific compounds, we employ differential screening. A concentrated fraction is applied to a lawn of E. coli, and, in parallel, to a plate with S. aureus that acts as a counterscreen. A fraction that has selective activity against E. coli is likely to be target specific so as to hit a target that is absent from Gram-positive bacteria. This approach is faster than engineering production but is relatively low throughput and is probably limited to producers, such as *Xenorhabdus* and *Photorhabdus*, that tend to make compounds that are of interest to us.

Using this approach, we identified ADG, which acts selectively against E. coli. ADG has been synthesized commercially as a potential antiviral (21). The fact that human chemists and nature came up with the exact same compound is a fascinating finding. Most of the work on nucleoside derivative prodrugs has been performed with antivirals such as AZT. All of these compounds are synthetic. Some of these antivirals (but not ADG) have been shown to act against bacteria, with genetic evidence pointing to the involvement of kinases (44, 45). No mechanism of action or targets have been described for bacteria. ADG is an analog of guanosine, and we find that in E. coli it is phosphorylated by enzymes of the purine salvage pathway: ADG (Gsk)–ADG-MP (Gmk)–ADG-DP (Ndk)–ADG-TP. The product, ADG-TP, is a mimic of GTP. ADG lacks the 3′-OH of guanine, and we determined that it interrupts RNA elongation. Remarkably, this is the same principle that was used by Sanger to interrupt DNA synthesis, which enabled sequencing. Again, human chemists and nature appear to have arrived at a similar solution, in this case how to terminate nucleic acid synthesis.

We identified the BGC of ADG based on homology with the BGC of puromycin in *S. alboniger.* Even though this BGC shares homology with puromycin BGC, it codes for a compound that has an entirely unrelated mechanism of action. This finding suggests that even closely related BGCs can diverge to produce secondary metabolites with completely different functions.

Notably, we identified mutants resistant to ADG in the Gsk and Gmk kinases but not in the RNA polymerase or any other potential target. We also noticed that cells treated with ADG are elongated, suggesting that the compound is interfering with GTP-dependent polymerization of FtsZ. Indeed, the FtsZ septation ring does not form in the presence of ADG. In previous studies, guanosine analogs have been suggested as FtsZ inhibitors and potential antibiotics (46). For instance, bromoguanosine (BrGTP) has been shown to be a competitive inhibitor for GTP during FtsZ assembly (47). It is unclear how many of the 80-odd proteins that use GTP are inhibited by its mimic, but it is safe to expect that it is more than just RNA synthesis and FtsZ.

While we set out to find target-specific compounds, unexpectedly, we discovered that ADG is a prodrug whose active form hits more than one target. The selectivity of action comes not from the target but from a prodrug-activating enzyme, the Gsk kinase unique to some Gram-negative bacteria. In terms of utility, this is equivalent to target-specific action. Ectopic overexpression of Gsk strongly increases susceptibility of E. coli to ADG, and this experiment probably emulates natural conditions under which the purine salvage pathway is important. The natural environment of E. coli is the nutrient-poor colon, where Gsk is likely to be expressed. ADG is probably smuggled into the cell and accumulates in the cytoplasm by active transport. We did not identify any resistant mutants in a transporter, which is to be expected, given that there are 3 redundant nucleoside transporters in E. coli: NupC, NupG, and YegT (48). A particularly intriguing possibility is that under conditions where Gsk contributes to fitness, it would be very difficult to impossible for target bacteria to develop resistance to this compound due to its multitargeting.

Several prodrugs are important antibiotics, such as metronidazole (49), and compounds acting selectively against Mycobacterium tuberculosis, such as isoniazid, ethionamide (50), and PA-824 (51). The selectivity comes from the activating enzyme. For example, the broad-spectrum metronidazole (49) is reduced by bacterial nitroreductases into a generally reactive compound that hits unrelated targets. Our knowledge of natural compound prodrugs is sparse and the number of classes is small. Sideromycins are chimeric compounds where the active moiety is linked to a siderophore, which enables cell penetration, and the active compound is released upon hydrolysis (52). The best-studied sideromycin is albomycin, a ferrichrome-thioribosyl-pyrimidine that inhibits seryl-t-RNA synthetase. Mitomycin C is reduced inside cells into an active compound that forms adducts with DNA, but reduction is not specific to bacteria, and this compound is used as an anticancer agent (53). Azomycin is a nitroaromatic compound from which metronidazole was derived (54). Azomycin has not been studied in any detail, and we assume that it has the same mode of action as its synthetic analog. There are numerous natural nitroaromatics that may act similarly to azomycin-metronidazole. Not all nitroaromatics are prodrugs; for example, chloramphenicol is a specific inhibitor of translation. Since prodrugs are activated by enzymes that may not be essential, null mutations lead to high frequency of resistance. This is not a major problem for metronidazole, since there are more than one nitroreductases in the cell.

The discovery of ADG expands our knowledge of natural product prodrugs and suggests that this is only the first representative of a larger group of nucleoside triphosphate mimics.

## MATERIALS AND METHODS

### Screening conditions.

*Photorhabdus* and *Xenorhabdus* strains in this study came from two libraries. One library containing 35 strains was obtained from Toyoshi Yoshiga, Saga University, Japan. The second library includes 25 strains and was obtained from Heidi Goodrich-Blair from Wisconsin-Madison University. Strains were inoculated in 50-mL Falcon tubes that contained 10 mL Luria–Bertani (LB) broth and incubated overnight at 28°C with shaking at 200 rpm, diluted 1:100 in new Falcon tubes with 10 mL LB broth and tryptic soy broth (TSB), and incubated for 8 days at 28°C with shaking at 200 rpm. Following the removal of the cells by centrifuging at 12,000 × *g* for 10 min, 10-mL culture supernatants were collected, dried, and resuspended in 500 μL MilliQ water to obtain 20 times concentrated sample compared to initial supernatant. These samples were subjected to reverse-phase chromatography, and HPLC elution was fractionated using a C_18_ semipreparative column (XBridge BEH C_18_, 5 mm; 10 mm by 250 mm). HPLC conditions were the following: solvent A, MilliQ water and 0.1% (vol/vol) formic acid; solvent B, acetonitrile and 0.1% (vol/vol) formic acid. The initial concentration of 2% solvent B was maintained for 5 min, followed by a linear gradient to 50% over 33 min with a flow rate of 4 mL/min. Fractions were collected every minute. UV detection was monitored at 254 nm. Each fraction was dried down and resuspended in 50 μL of MilliQ water. Overlays were prepared from exponential cultures. After dilution of 1:100 from an overnight culture in cation-adjusted Mueller-Hinton II broth (MHIIB), test pathogens were grown for 2 to 5 h at 37°C with shaking at 220 rpm and diluted to an optical density at 600 nm (OD_600_) of 0.03 in MHIIB. Cation-adjusted Mueller-Hinton II agar (MHIIA) plates were covered by these cultures, and the excess culture was removed and overlays were left to dry in a biosafety cabinet. Fractions were tested for activity by spotting 6 μL on Mueller-Hinton II agar lawns that were seeded with S. aureus HG003 as a representative of Gram-positive bacteria and E. coli MG1655 as a representative of Gram-negative bacteria for evaluating the activity.

### Strain fermentation and purification of ADG.

*P. luminescens* KLE11358 was inoculated in a 250-mL Erlenmeyer flask with 100 mL LB broth and incubated at 28°C with shaking at 200 rpm. Following overnight incubation, it was diluted 1:100 into a 2-liter Erlenmeyer flask with 1 liter tryptic soy broth and incubated for 8 days at 28°C with shaking at 200 rpm. Cells were removed by centrifugation at 8,000 × *g* for 10 min, and XAD16N resin (20 to 60 mesh; Sigma-Aldrich) was added to the culture supernatant at 15% (vol/vol) and incubated overnight under agitation to bind ADG. ADG was eluted from the XAD16N resin using 1 liter of 100% methanol. The eluate was concentrated using a rotary evaporator. The solution was subjected to HPLC fractionation at the preparative scale using a C_18_ column (Luna C_18_, 5 mm; 250 by 21.2 mm). HPLC conditions were solvent A, MilliQ water and 0.1% (vol/vol) formic acid; solvent B, acetonitrile and 0.1% (vol/vol) formic acid. The initial concentration of 2% solvent B was maintained for 10 min, followed by a linear gradient to 50% over 43 min with a flow rate of 7 mL/min. Fractions were collected every minute. UV detection was monitored at 254 nm. Active fractions from the previous step were combined and subjected to further fractionation by using C_18_ semipreparative column (XBridge BEH C_18_, 5 mm; 10 mm by 250 mm). HPLC conditions were solvent A, 50 mM ammonium acetate pH 8; solvent B, acetonitrile. The initial concentration of 2% solvent B was maintained for 3 min, followed by a linear gradient to 40% over 8 min with a flow rate of 5 mL/min. UV detection was monitored at 254 nm. Bioactivity assay led to detection of the active peak. For final purification, active fraction from the previous step was subjected to HPLC using C_18_ semipreparative column (XBridge BEH C_18_, 5 mm; 10 mm by 250 mm). HPLC conditions were solvent A, MilliQ water and 0.1% (vol/vol) formic acid; solvent B, acetonitrile and 0.1% (vol/vol) formic acid. The initial concentration of 1% solvent B was maintained for 2 min, followed by a linear gradient to 2.2% over 8 min with a flow rate of 5 mL/min. UV detection was monitored at 254 nm. ADG elutes at 5.57 min (see Fig. S1a in the supplemental material).

### LC-MS analysis.

LC-MS analysis was conducted on an Agilent HPLC 1260 Infinity II coupled with an Agilent 6530 Q-TOF-LC-MS system (Agilent Technologies, Palo Alto, CA, USA). The HPLC column was a reversed-phase ZORBAX RRHT Extend-C_18_, 2.1 by 50 mm, 1.8 μm (Agilent Technologies, USA). The mobile phase was a gradient of 0.1% formic acid in water (A) and acetonitrile (B) at a flow rate of 0.2 mL/min. A linear gradient was initiated with 2% acetonitrile and linearly increased to 95% at 5.1 to 10 min. The flow rate was 0.2 mL/min, and the injection volume was 5 μL. Mass spectra in the *m/z* range 111 to 3,000 were obtained by positive ion (positive electrospray ionization) modes. The mass spectrometry conditions were the following: gas temperature, 300°C; N2 flow rate, 7 liters/min; nebulizer gas pressure, 35 lb/in^2^; capillary voltage, 3,500 V; fragmentor potentials, 175 V; Vcap, 3,500 V; skimmer, 65 V; and octopole RFPeak, 750 V. Data acquisition and analysis were conducted using Agilent LC-MS-QTOF MassHunter Data Acquisition Software version 10.1 and Agilent MassHunter Qualitative Analysis Software version 10.0, respectively (Agilent Technologies, USA).

### Structure determination of ADG.

3′-Amino-3′-deoxyguanosine (ADG): white powder, HRESIMS *m/z* 283.11 [M+H]^+^ (calculated for C_10_H_15_N_6_O_4_^+^, 283.1149); ^1^H-NMR (500 MHz, D2O) *δ* 8.03 (s, 1H), 5.98 (d, *J *=* *2.6 Hz, 1H), 4.64 (br s, 1H), 4.10 (br s, 1H), 3.99 (dd, *J *=* *12.8, 2.1 Hz, 1H), 3.84 (dd, *J *=* *12.8, 4.2 Hz, 1H), 3.68 (t, *J *=* *6.2 Hz, 1H); ^1^H-NMR (100 MHz, DMSO) *δ* 157.4, 154.3, 150.8, 135.0, 116.6, 88.1, 85.1, 75.0, 61.0, 52.6.

The ^1^H-NMR spectrum of ADG in D_2_O showed one singlet aromatic proton (*δ*H 8.03), one doublet anomeric proton (*δ*H 5.98, *J *=* *2.6 Hz), and five signals at *δ*H4.64 to 3.68, suggesting the presence of a sugar unit (Fig. S2a). One spin system from the anomeric proton (*δ*H 5.98) through three methines (*δ*H 4.64, 3.68, and 4.10) to the oxymethylene (*δ*H 3.99 and 3.84) observed in ^1^H-^1^H correlation spectroscopy (COSY) spectrum established the existence of a furanose unit (Fig. S2b). The guanine moiety was then deduced based on the following observations. (i) One diagnostic singlet proton at *δ*H 8.03 was observed in the ^1^H-NMR spectrum. (ii) The UV spectrum showed the characteristic absorption maxima at 255 and 275 nm (Fig. S4b, left). (iii) ADG was poorly retained on C_18_ reversed-phase HPLC column. Considering the similar molecular mass with nucleoside guanosine (283.241 g/mol), together with the evidence described above, the compound was deduced to be the derivative of guanosine. The upfield ^1^H-NMR signal (*δ*H 3.68) indicated that the methine group at C-3′ in the furanose unit is replaced by the amine group rather than the hygroxy group (Fig. S2d). This conclusion was further supported by the ^13^C NMR spectrum of ADG comparable to that of adenosine except for the presence of upfield signal (*δ*C 52.6) (Fig. S2c). Therefore, the structure of the compound was determined as a derivative of 3′-deoxyguanosine aminated at C-3′. The resulting structure of the ADG was also confirmed by comparison of the ^1^H-NMR spectrum with that of the authentic synthetic sample obtained from BIOSYNTH Carbosynth (Reading, UK). A mixture of two compounds displayed only one set of ^1^H-NMR signals (Fig. S4a). The synthetic sample showed potency (MIC) and spectrum of activity identical to those of the natural ADG (Table S2).

10.1128/mbio.00700-22.8 TABLE S2 Minimal inhibitory concentrations for natural ADG and synthetic ADG. Download Table S2, DOCX file, 0.06 MB. Copyright © 2022 Shahsavari et al. 2022 Shahsavari et al. https://creativecommons.org/licenses/by/4.0/ This content is distributed under the terms of the Creative Commons Attribution 4.0 International license.

### Identification of the putative BGC.

Assembly of Illumina genome sequencing of *P. luminescens* WM06 data using Spades (55) resulted in a 4,919,445-bp genome with a GC content of 43%. MultigeneBlast (56) of the Purimycin BGC from *S. alboniger* (GenBank accession no. X92429.1) was performed against the producer strain genome, and a small 5-gene BGC candidate consisting genes homologous to *pur10*, *pur4*, *pur7*, and *pur3* as well as a gene annotated as an MFS transporter gene were identified (Fig. 1b and Table S3).

### Heterologous expression.

To prove the putative BGC involvement in ADG biosynthesis, the BGC was heterologously expressed in E. coli Bap1. Genomic DNA of the ADG producer strain was isolated using the Qiagen blood and tissue kit (Qiagen, Germany) according to manual, and the whole BGC (omitting the MFS transporter gene) was amplified with matching overlap using the primers 5′-GTTAAGTATAAGAAGGAGATATACAATGAAGCTGGTTATAATTGGTTGCG-3′/5′-TGCTCAGCGGTGGCAGCAGCCTATGAATTTAGCGAAGCTATAACCGAATC-3′ and Q5 DNA polymerase (NEB Biolabs, USA) according to the manufacturer’s instructions. Simultaneously, the vector pRSFduett-1 (Invitrogen) was amplified with the primers 5′-GCTGCTGCCACCGCTGAGCA-3′/5′-TGTATATCTCCTTCTTATACTTAACTAATATACTAAGATGGG-3′ using the Q5 DNA polymerase according to the manual, and both fragments were gel purified on 1% Tris-acetate-EDTA (TAE) agarose gels. DNA was recovered from the gels using the large-fragment DNA recovery kit (ZymoResearch, USA), and both fragments were fused using NEB isothermal assembly mix (NEB Biolabs, USA). E. coli DH5α was transformed with the isothermal assembly reaction using standard methodology and selected on LBKan agar plates. Correct assembly was verified by test restriction using EcoRI*/*SwaI, and the plasmid pNS-ADG was introduced to E. coli Bap1 using standard electroporation transformation methodology. E. coli Bap1+pNS-ADG and E. coli Bap1+pRSFduett-1, serving as a negative control, were grown in 1 liter LBKan at 37°C and 200 rpm to an OD_600_ of ~0.5. Expression of the genes was induced by addition of IPTG to 1 mM, and incubation was continued at 30°C and 200 rpm. After 3 days, cells were removed by centrifugation at 8,000 × *g* for 10 min, and XAD16N resin (20 to 60 mesh; Sigma-Aldrich) was added to the culture supernatants at 15% (vol/vol) and incubated overnight under agitation to bind. Methanol (100%, 1 liter) was used to elute the resin. The eluate was concentrated using a rotary evaporator redissolved in water and subjected to C_18_ solid-phase extraction. The medium was desalted by application of 6 column volumes (CV) MilliQ water and eluted with 6 CV 80% acetonitrile plus 0.1% formic acid. Elution fractions were evaporated to dryness using a SpeedVac system. The solutions were then subjected to HPLC fractionation using C_18_ column (XBridge BEH C_18_, 5 mm; 10 mm by 250 mm). HPLC conditions were solvent A, MilliQ water and 0.1% (vol/vol) formic acid; solvent B, acetonitrile and 0.1% (vol/vol) formic acid. The initial concentration of 1% solvent B was maintained for 5 min, followed by a linear gradient to 10% over 37 min with a flow rate of 5 mL/min. Fractions were collected from 5.5 min to 6.5 min based on the elution time of ADG standard (100 μg/mL) under this HPLC condition. Samples were dried, redissolved in water, and subjected to LC-MS analysis.

### MIC assays and cytotoxicity.

The MIC was determined by a broth microdilution assay. Overnight cultures of E. coli strains, P. aeruginosa PAO1, A. baumannii ATCC 17978, K. pneumoniae strains, and S. aureus HG003 were diluted 1:100 in MHIIB and incubated at 37°C with aeration at 220 rpm. Exponential cultures with an OD_600_ of 0.1 to 0.9 were diluted to an OD_600_ of 0.001 in MHIIB; 98-μL aliquots were transferred into round-bottom 96-well plates containing 2 μL of ADG solutions with 2-fold serial dilution. After overnight incubation at 37°C, the ADG MIC was determined as the minimum concentration at which no growth of strains could be detected by eye. The MIC against intestinal bacteria, Klebsiella variicola KLE 2552, Veillonella ratti KLE 2365, Clostridium bifermentans KLE 2329, Clostridium hylemonae KLE 2503, Escherichia fergusonii KLE 2502, Enterococcus faecalis KLE 2341, and Stenotrophomonas maltophilia KLE 11416 (KLE collection bacteria were isolated from stool under anaerobic conditions and identified by 16S sequencing), was determined under anaerobic conditions (Coy vinyl anaerobic chamber, 37°C, 5% H_2_, 10% CO_2_, 85% N_2_). Overnight cultures grown in brain heart infusion (BHI) broth, supplemented with 0.5% yeast extract, 0.1% l-cysteine hydrochloride, and 15 μg/mL hemin (BHI-Ych), were diluted 1:100 in BHI-Ych. The 96-well assay plates were prepared by 2-fold dilution of ADG and included a positive growth control. After 24 h of incubation, the MIC was determined. All MIC assays were performed at least in triplicate. Cytotoxicity assay with FaDu and HepG2 cell lines was conducted as previously mentioned (18).

### Resistance studies.

Three independent colonies were used for each resistance study. For E. coli MG1655 wild-type strain, exponential cultures were washed in phosphate-buffered saline (PBS) and subsequently inoculated onto 25-mL MHIIA plates containing 4× MIC ADG at a density of 1.8 × 10^6^ CFU per plate. For E. coli *gsk++* strain, exponential cultures with a density of 1.5 × 10^9^ CFU per plate were washed in PBS and subsequently inoculated onto 25-mL MHIIA plates containing 16× MIC ADG. After 48 h of incubation the plates were examined for colonies, and the number of colonies was counted. The colonies were restreaked to test for resistance stability. Genome sequencing and variant calling were conducted by the Microbial Genome Sequencing Center (MiGS, Pittsburg, PA). Whole-genome sequencing was performed by paired-end reads (2 × 150 bp) with Illumina NextSeq 550, and *E. coli* genome information data in NCBI (GenBank accession no. U00096.2) was used for variant calling. GSK-F primer (TTCGCCGCTCAGTTAACCAC) and GSK-R primer (AGGCATCGAGAGCCAAATGC) were used to amplify *gsk* in resistant mutants and verify the mutations from Illumina whole-genome sequencing. Primers up-gmk-f (CAGTGAATGACAGGCAAATGC) and down-gmk-r (CCTACCTGCATCTGACGAGC) were used to amplify *gmk* and verify the mutations from Illumina whole-genome sequencing in resistant mutants from *gsk++* strain.

### Fluorescence microscopy.

For time-lapse microscopy, E. coli MG1655 was cultured in MHIIB overnight to stationary phase and the following day was inoculated into fresh MHIIB at 1:100 and grown for 2 h at 37°C. Cells were diluted 10-fold in MHIIB, placed on top of a 1.5% low-melting-point agarose MHIIB pad containing ADG (128 μg/mL) and dyes Sytox Green (0.5 μM) and FM4-64 (10 μg/mL) from Molecular Probes, and observed with a Nikon Ti2-E fluorescence microscope using a 100× oil-immersion lens objective. The fluorescence signals for Sytox Green and FM4-64 were collected sequentially by excitation at 480 nm and 550 nm, respectively, alongside a phase-contrast image. A thermostatic chamber was used to maintain a temperature of 37°C for the duration of the experiment. Images were acquired with NIS-Elements every 15 min at a resolution of 2,048 by 2,048. For FtsZ localization, E. coli MG1655 FtsZ-GFP (gift from Thomas Bernhardt, Harvard Medical School) was grown as outlined above. After the 2-h outgrowth, 1-mL aliquots were taken and treated with ADG (128 μg/mL), ciprofloxacin (1 μg/mL), cephalexin (8 μg/mL), or no drug for 1 h at 37°C. Cells were spotted onto a 1.5% low-melting-point agarose pad and observed with a Nikon Ti2-E fluorescence microscope using a 100× oil-immersion lens objective. Fluorescence signal from FtsZ-GFP was collected by excitation at 480 nm alongside a phase-contrast image. All images were processed with Fiji software (57). For visual representation in Fig. 5, background was subtracted (rolling ball radius of 50 pixels) for the fluorescent channel, and brightness/contrast was further adjusted relative to nonfluorescent E. coli MG1655. The HyperStackReg plugin was utilized to correct the *x*/*y* drift in Video S1.

### Construction of the E. coli *gsk*++ strain.

To insert a second copy of *gsk* in the chromosome of E. coli MG1655, the locus of *gsk* was fused to a kanamycin resistance marker, and this DNA product was inserted into the neutral *fliT* site (24) through λ Red recombination (25). More precisely, *gsk* was amplified by PCR using the primers fliT-gsK-F (5′-GCAAGGTAAAGCAGTTATTACAGATTCGGATGGATGAACTGGCGAAACTGTCGTTGCTCAG CAATCG-3′) and gsk-kan-R (5′-GAAGCAGCTCCAGCCTACAACGGTGGTTGCCGGATGTAAAATATG-3′) with the genomic DNA of E. coli MG1655 as the template, and the kanamycin resistance marker was amplified using the primers gsk-kan-F (5′-CATATTTTACATCCGGCAACCACCGTTGTAGGCTGGAGCTGCTTC-3′) and fliT-kan-R (5′-CAGCGTGGGATTACGCATTCTTCGACTCCATTCAAGGGGAACATTAGAAGAATTAGCCATGGTCCATATG-3′) with an in-house-modified plasmid as the template. Those two DNA products were then fused by PCR using the primer pair fliT-gsk-F/fliT-kan-R. Approximately 500 ng of this column-purified DNA product was used to transform electrocompetent cells of E. coli MG1655-pKD46 to perform λ Red recombination. The subsequent steps have been adapted from the Quick and Easy E. coli gene deletion kit (GeneBridges, Heidelberg, Germany). Briefly, 30 μL of an overnight culture of E. coli MG1655-pKD46 was used to inoculate a microtube containing 1.4 mL of LB complemented with ampicillin (100 μg/mL). After 2 h of shaking at 30°C, 0.4% of l-arabinose was added, and the tube was transferred for shaking at 37°C for 1 h. Cells were washed and concentrated with ice-cold 10% glycerol prior to electroporation. The recovery step was performed for 3 h at 37°C with shaking. The selection was performed using resistance to kanamycin (50 μg/mL). Several transformant clones were then restreaked with double selection for resistance to kanamycin (50 μg/mL) and sensitivity to ampicillin (100 μg/mL at 30°C). The neutral *fliT* site was amplified using the primers fliT-up (5′-CAACGGAAGAACAATGGGAC-3′) and fliT-down (5′-TCATAGGAGTCGTAGCCGAC-3′) and sequenced, and the presence of *gsk* within this locus was confirmed.

### Subcloning.

The E. coli *gsk* open reading frame (ORF; stop codon included) was subcloned into pET Sumo vector (Invitrogen) immediately downstream to the 5′ end of the His6-Sumo ORF (58). The E. coli *gmk* and *ndk* ORFs were subcloned into pET28b vector between NdeI and XhoI restriction sites (59).

### Recombinant proteins.

All three recombinant proteins, namely, His6-Sumo-Gsk, His6-Gmk, and His6-ndk, were expressed in E. coli BL21(DE3) harboring the corresponding expression plasmid. Expression strains were grown in LB containing 30 μg/mL kanamycin at 37°C to an OD_600_ of 0.6. The culture was then cooled to 18°C and induced by the addition of 200 μM IPTG. Cells were harvested 16 h postinduction. The cell pellet from 1 liter of expression culture was resuspended in 25 mL lysis buffer containing 50 mM Tris-HCl, pH 8.0, 150 mM KCl, 1 mM Tris(2-carboxyethyl)phosphine hydrochloride (TCEP), 20 μg/mL lysozyme, 10 U Benzonase, and 1 mM phenylmethylsulfonyl fluoride (PMSF). Cells were disrupted through sonication and the lysate was cleared at 12,000 × *g* for 1 h. Cleared lysate was applied to 1 mL Ni-NTA resin equilibrated with the lysis buffer and allowed to flow through by gravity. The Ni-NTA resin was washed with 5× column volumes of wash buffer 1 (50 mM Tris-HCl, pH 8.0, 500 mM KCl, 10 mM imidazole, and 1 mM TCEP) and 10× column volumes of wash buffer 2 (50 mM Tris-HCl, pH 8.0, 150 mM KCl, 25 mM imidazole, 1 mM TCEP). Bound protein was eluted with 3× column volumes of elution buffer (50 mM Tris-HCl, pH 8.0, 150 mM KCl, 300 mM imidazole, and 1 mM TCEP). Eluates of the Ni-NTA column were dialyzed against 20 mM Tris-HCl, pH 8.0, and 150 mM KCl. His6-Sumo-Gsk then was treated with the Sumo protease, Ulp1 (10 μg per μmol cleavage sites), in the presence of 1 mM TCEP, and His6-Gmk and His6-Ndk with thrombin (10 U per μmol cleavage sites), both at room temperature overnight. Cleavage mixtures were concentrated and Gsk, Gmk, or Ndk was further purified over a Superdex-200 10/300 column run in 20 mM HEPES-Na, pH 7.0, 150 mM KCl (for Gsk) or NaCl (for Ndk and Gmk), and 1 mM TCEP.

### Kinetics of ADG phosphorylation by Gsk.

All nucleotides were quantified using absorbance spectrometry. The wavelength was 251 nm for guanosine and ADG nucleotides and 260 nm for adenosine nucleotides; extinction coefficients were 13.6 × 10^3^ M^−1^ cm^−1^ and 15.4 × 10^3^ M^−1^ cm^−1^, respectively. For real-time monitoring of the reaction progress, ADP produced from Gsk activity was coupled to the consumption of NADH by the activities of pyruvate kinase (PK) and lactate dehydrogenase (LDH). The overall reaction was

$$\text{ADG }\left(\text{or guanosine}\right)\;+\text{ PEP }+\text{ NADH }+{\text{ H}}^{+}\;\to \text{ ADG}-\text{MP }\left(\text{or GMP}\right)\;+\text{ lactate }+{\text{ NAD}}^{+}$$

All reactions were performed at 100-μL scale in 96-well plates at 30°C in a SpectraMax M5 plate reader (Molecular Devices). Absorbance of each reaction was monitored for the absorbance at 340 nm (*A*_340_) every 30 s. ADG and guanosine were each dissolved in water at 100 mM in the presence of equimolar NaOH. ATP was dissolved in water at 100 mM and pH adjusted to 7.4.

A 10× pyruvate kinase-lactose dehydrogenase (PK-LDH) mixture was prepared in water that contained phosphoenolpyruvate (10108294001, 37.5 mM; PEP; Roche), NADH (10107735001, 7.5 mM; Roche), PK (P9135; Sigma), and LDH (10127230001, 100 U/mL each; Roche) (60). Final reaction mixtures contained 50 nM Gsk, 5 mM ATP, 1 mM ADG or guanosine in reaction buffer (40 mM HEPES-Na, pH 7.4, 150 mM KCl, 10 mM MgCl_2_, and 1 mM TCEP). The reaction was assembled by first mixing all components except ADG or guanosine. This master mix and nucleoside substrates were predeposited in separate wells on the same plate. After preincubating the plate at 30°C for 5 min, the mixture was then transferred to the substrate using a multichannel pipette at *t* = 0 to initiate the reaction. The initial velocity (in *A*_340_/min) was calculated by linear regression of the initial linear segment of each kinetic curve.

### Detection of ADG-MP, ADG-DP, and ADG-TP by anion-exchange chromatography.

All reactions were performed at 100-μL scale in Eppendorf tubes. Final reaction mixtures contained 1 μM Gsk and 2 mM ATP in reaction buffer (40 mM HEPES-Na, pH 7.4, 150 mM KCl, 10 mM MgCl_2_, and 1 mM TCEP). ADG (1 mM), Gmk (1 μM), Ndk (1 μM), PEP (4 mM), and PK (10 U/mL) were also included as indicated. The reaction mixture was incubated at 37°C for 30 min and then quenched by addition of 100 μL chloroform and vortexing. Following centrifugation at 8,000 × *g* for 5 min, 2 μL aqueous phase was diluted in 200 μL 5 mM Tris-HCl, pH 8.0, and applied to MonoQ 5/50 running at 1 mL/min with buffer A (5 mM Tris-HCl, pH 8.0). Nucleotides were resolved using a linear gradient in which percentage of buffer B (5 mM Tris-HCl, pH 8.0, 1 M NaCl) increased from 0% to 40% in 20 mL, starting at *V* = 2.5 mL.

### Scaled-up synthesis of ADG-TP.

In a 15-mL Falcon tube, ADG (14.1 mg, 50 μmol) was solubilized in 0.5 mL 100 mM NaOH and then diluted in 4 mL reaction buffer. To the solution was added ATP (50 μL of 100 mM solution adjusted to pH 7.4, 5 μmol, 0.1 eq) and PEP (440 μL 400 mM solution adjusted to pH 7.4, 176 μmol, 3.5 eq). Gsk, Gmk, and Ndk then were added to a final concentration of 3 μM and PK to 10 U/mL. The reaction was expanded to 5 mL using reaction buffer, mixed thoroughly, and incubated at 37°C for 2 h. Analysis using MonoQ 5/50 showed that ADG was completely converted to ADG-TP. ADP was then carefully titrated in to remove excess PEP, which would otherwise be difficult to separate from ADG-TP. Nevertheless, excess ADP would cause Ndk-dependent dephosphorylation of ADG-TP and therefore must be avoided. The reaction was then quenched by addition of 2 mL chloroform and vortexing. Following centrifugation at 8,000 × *g* for 5 min, the clear, upper, aqueous layer was transferred to a fresh Falcon tube and subjected to vacuum to remove residual chloroform. The reaction was further treated with Tas1 (10 nM final concentration [61] at 37°C for 1 h to eliminate ATP, which would otherwise be difficult to separate from ADG-TP). The reaction was then diluted to 50 mL with water and ADG-TP was purified over MonoQ 10/100 in two parallel runs using a linear gradient in which percentage of buffer B increased from 5% to 30% in 200 mL. Lithium chloride (1.0 M) was added to the combined eluate containing pure ADG-TP, and ADG-TP was precipitated by the addition of ethanol to a final concentration of 80% (vol/wt). The precipitate was collected by centrifugation at 8,000 × *g* for 5 min, washed once with 95% ethanol, and then dissolved in water. This solution, containing 35.5 μmol (71%) ADG-TP, was lyophilized and redissolved in water for further use. MALDI-TOF [M+H]^+^ *m/z* calculated for C_10_H_18_N_6_O_13_P_3_, 523.011; found, 523.141.

### *In vitro* transcription assay.

MEGAscript T7 transcription kit was purchased to perform the *in vitro* transcription assay, and the assay was carried out according to the kit instruction manual. The frozen reagents were thawed, and the transcription reaction mixture consisting of 2 μL of each nucleotide (ATP, GTP, UTP, and CTP), 2 μL of the reaction buffer, 2 μL of the enzyme mix, 5 μL nuclease-free water, and 1 μL DNA template (the DNA template that was included in the kit was diluted 5-fold and used for this study) were assembled at room temperature. A volume of 18 μL of reaction mixture was moved into 1.8-mL Eppendorf tubes containing 2 μL of ADG-TP solutions with 2-fold serial dilution and one tube containing 2 μL of RNase-free water as a negative control. After mixing thoroughly, the reaction solutions were incubated at 37°C for 4 h. Following incubation, the reaction solutions were mixed with gel loading buffer II at a 1-to-1 volume ratio and loaded on TAE agarose gel containing 0.5 μg/mL ethidium bromide for electrophoresis.

Templates for the synthesis of AAG and AAAG were fully complementary, double-stranded DNA. Coding strand sequences are '5-CAGTAATACGACTCACTATT**AAG**CCTCGCTC-3′ and 5′-CAGTAATACGACTCACTATT**AAGA**CCTCGCTC-3′, respectively. Note that CTP was not added and therefore downstream sequences are not transcribed. DNA oligonucleotides were first annealed in 10 mM Tris-HCl, pH 8.0, 50 mM NaCl, and 0.1 mM EDTA using a temperature program for 2 min each incubation at 80°C, 75°C, 70°C, 65°C, 60°C, 55°C, 50°C, 45°C, and 40°C (in this order). Transcription was carried out using 6 mM ATP, 2 mM GTP (or ADG-TP), 1 μM double-stranded template, and 1 μM T7-RNA polymerase in the presence of 100 mM Tris-HCl, pH 8.0, 20 mM MgCl_2_, 2 mM spermidine trihydrochloride, and 20 mM dithiothreitol (DTT). Following incubation at 37°C for 40 min, a 5-μL aliquot was withdrawn from the cloudy suspension, diluted in 0.5 mL cold water, and applied to a MonoQ 5/50 column. Bound nucleotide was eluted using a gradient with the percentage of buffer B increasing from 15% to 36% in 14 mL. To prepare AAG, AAGA, and AA(ADG) for mass spectrometry, 100 μL of the appropriate reaction mix was diluted in 0.9 mL cold water and filtered using an Amicon Ultra 0.5 concentrator with a 10-kDa molecular mass cutoff. The filtrate was applied to a MonoQ 5/50 column and fractionated using a gradient with the percentage of buffer B increasing from 10% to 30% in 20 mL. Volumes of 200 μL from peak fractions containing pppAAG, pppAAGA, and pppAA(ADG) were each treated with 1 μL Quick CIP (NEB) at 37°C for 30 h, and the oligoribonucleotides with 5′-OH were acidified using 10 mM HCl and applied to a Discovery Wide-Pore C_18_ (Supelco) column (150 by 4.6 mm, 5 μm) using solvents A (0.1% trifluoroacetic acid [TFA] in water) and B (0.1% TFA in 90:10 acetonitrile-water). All nucleotides were eluted using a gradient with the percentage of solvent B increasing from 0% to 30% in 10 mL. Fractions containing each nucleotide were lyophilized and dissolved in 0.1% formic acid in water for *m/z* analysis using a Sciex X500B QTOF mass spectrometer. The mass spectrometer was controlled by Sciex OS v.1.6.1 using the following settings: ion source gas 1, 30 lb/in^2^; ion source gas 2, 30 lb/in^2^; curtain gas, 35; CAD gas, 7; temperature, 300°C; spray voltage, −4,500 V; declustering potential, −80 V; collision energy, −10 V. Data were acquired from 400 to 2,000 Da with a 0.5-s accumulation time and 4 time bins summed.

### Metabolomics studies.

Overnight cultures of E. coli MG1655 from three independent colonies were diluted 1:10,000 into 100 mL MHIIB medium in 250-mL Erlenmeyer flasks and incubated at 37°C with shaking at 220 rpm. After 4 h, 10 mL from each flask was collected (and kept on ice), OD was measured, and ADG was added to the cultures at 1× MIC. After 1 h of incubation, 10 mL from each flask again was collected (and kept on ice) and OD was measured. To normalize the amount of metabolome collected before and after treatment with ADG, OD × volume = 2.5 was used. Cells from each sample were collected using a PVDF membrane and a vacuum filter. The membranes then were moved in a 20-mL glass vial containing 4 mL methanol and vortexed for 1 min to lyse the cells. The membranes then were removed, and 8 mL of chloroform was added, followed by vortexing for 1 min and incubating in an ultrasound bath for 5 min; 4 mL of water containing 0.05 μM internal standards (guanosine-^15^N_5_ 5′-monophosphate [900380; Sigma-Aldrich], adenosine-^15^N_5_ 5′-diphosphate [741167; Sigma-Aldrich], and guanosine-^13^C_10_ 5′-triphosphate [710687; Sigma-Aldrich]) were added, followed by vortexing for 1 min and incubating in an ultrasound bath for 5 min. The 20-mL vials were placed in 50-mL centrifuge tubes and centrifuged for 10 min at about 3,000 rpm to achieve phase separation. Next, the top layer (7.5 mL, aqueous phase) was collected and dried using a SpeedVac system. Samples were resuspended in 100 μL of MilliQ water and submitted to the Harvard Center for Mass Spectrometry for measurements. All samples were run on a ThermoFisher IDX using a HILICON column (iHILIC-P Classic column, 150 by 2.1 mm by 5 μm). LC method was solvent A, 20 mM ammonium carbonate, 0.1% ammonium hydroxide, in water; solvent B, acetonitrile 97%, in water. The LC was equilibrated with 100% solvent B, and the initial concentration of 100% solvent B was decreased by a linear gradient to 40% over 19 min followed by 100% solvent A for 5 min with a flow rate of 0.15 mL/min. Peaks of ADG ([M+H]^+^ = 283.1149 and [M-H]^−^ = 281.1004) and candidate peaks of ADG-MP ([M+H]^+^ = 363.0813 and [M-H]^−^ = 361.0667), ADG-DP ([M+H]^+^ = 443.0476 and [M-H]^−^ = 441.033), and ADG-TP ([M+H]^+^ = 523.0139 and [M-H]^−^ = 520.9994) were integrated for all samples. The ratios of the targeted peak areas divided by the standard peak (IS) were calculated.

### Macromolecular label incorporation assay.

Macromolecular incorporation assay was performed as a service by ImQuest Biosciences. E. coli W0153 strain and ADG at 2× MIC were used in this study; 48 h prior to the assay, E. coli W0153 strain was inoculated onto Trypticase soy agar (TSA) with 5% sheep blood (SB) and incubated at 37°C. One day prior to assay initiation, 3 to 4 colonies from the agar plate were restreaked onto another TSA plus 5% SB and incubated overnight at 37°C. On the day of assay, 5 to 7 colonies from the overnight plate were inoculated into TSB and incubated at 37°C with shaking to an OD_600_ of 0.3 to 0.4. The cultures then were centrifuged, resuspended in fresh TSB or M5T broth (protein synthesis), and adjusted to an OD_600_ equivalent to 0.3. The impact of ADG was evaluated at 2× MIC. ADG and controls (ciprofloxacin, rifampicin, chloramphenicol, and triclosan) were diluted to 2 times the final in-well concentration and distributed in triplicate in a volume of 50 μL to wells of a 96-well round-bottomed microtiter plate; 50 μL of bacteria at an OD_600_ of 0.3 containing the appropriate amount of radiolabeled precursor ([^14^C]thymidine, 0.4 μCi/mL; [^14^C]uridine, 0.2 μCi/mL; [^14^C]l-amino acid mixture, 0.75 μCi/mL; and [^14^C]acetic acid, 6.0 μCi/mL) were added to the wells containing the compounds and incubated for 30 min at 37°C with shaking. Following the incubation, 100 μL of cold 10% trichloroacetic acid (TCA) was added to the wells of the 96-well plate containing the mixture. The plate was incubated for an additional hour on ice to allow for the radiolabeled precursor-incorporated material to precipitate. Following the incubation, all contents in the 96-well microtiter plate were transferred to a filter plate, and the nonprecipitated radioactive precursor was vacuum filtrated. Each well of the filter plate was washed three times with 200 μL of cold 5% TCA followed by three times with 200 μL of water using vacuum filtration. The filters were transferred to a Microbeta cassette, and 25 μL of scintillation fluid was added to each well. The plate was sealed, and each well was counted on a Perkin Elmer Microbeta scintillation counter.

### Predictive modeling of ADG mechanism of action.

Escherichia coli strain W0153 (parent strain AB1157; *asmB1* Δ*tolC*::*kan* modifications) was acquired from the Yale culture collection (http://cgsc2.biology.yale.edu/Strain.php?ID=4509) and previously calibrated using explainable artificial intelligence as a bioreporter for known MOAs (27). For the antibiotic challenge, 3 mL of E. coli strain W0153 at an OD_600_ of 0.5, representing mid-log phase, was exposed in biological triplicate at 1× MIC for 30 min. After 30 min of exposure, 100 μl of the cells was removed for OD_600_ values and CFU counts/mL. This served as a checkpoint to observe that the 1× MIC antibiotic treated sample is showing an OD_600_ value and CFU counts/mL less than that of the untreated control, *t *=* *30 min solvent control, but greater than that of the *t *=* *0 sample, to ensure proper growth and to rule out an overtreatment of the cells for an incorrect MIC. In parallel, the remainder of the cells was immediately pelleted at 4°C by centrifugation for 10 min at 2,000 rpm in 1-mL aliquots. The supernatant was removed, and the pellets were immediately frozen in liquid nitrogen and stored for the RNA extraction processing at a later date. Total RNA was extracted by automation using the NucleoMag RNA extraction kit (Macherey-Nagel, GmbH) on the EpMotion Robotic liquid handler. For the resulting total RNA, RNA integrity number (RIN) values were obtained to check for RNA quality using the 2200 TapeStation (Agilent Genomics, Inc.). Acceptable values to proceed to ribosomal subtraction were above a RIN of 5. rRNA was subtracted from the total RNA to yield only mRNA for library construction using a bacterial rRNA depletion kit (New England Biolabs, Inc.) at half reactions with a total RNA input maximum of 400 ng. The rRNA depleted product was quality controlled using an Agilent Bioanalyzer with the Agilent Pico chip for RNA detection to check for less than 0.5% of rRNA remaining; 2.5 μL of the rRNA-depleted samples, amounting to approximately 2 to 5 ng, is used as the input material to construct each cDNA library for RNA sequencing using the NEBNext ultradirectional RNA library prep kit (Illumina, Inc.) at half reactions. The resulting libraries were analyzed using Agilent high-sensitivity DNA chips to ensure library quality. Libraries were quantified and normalized by qPCR and then we sequenced, using the NovaSeq platform, approximately 9 million 75-bp paired-end reads for each library. To identify the primary MOA of ADG, transcriptomes from E. coli W0153 challenged with ADG were evaluated by the CoHEC predictive model (v7.0) and training data from Espinoza-Dupont et al. (27) using the Soothsayer (v2022.01.07) package in Python (https://github.com/jolespin/soothsayer). The predictive model was trained on 41 known antibiotic compounds representing 6 primary MOA containing a mixture of pure compounds and producer-strain extracts. This model was previously used (27) to flag darobactin as having novel activity based on standard error profiles, and the same approach was applied to ADG transcriptomes at 1× MIC/30 min of exposure. That is, the model was trained on the 41 known antibiotics and used to predict the MOA of ADG while using the replicates to calculate standard errors. The unsupervised clustering was performed using leave-compound-out cross validation where the test compound was held out during model fitting, the transcriptomes from the test compound were then evaluated by the model, and the resulting probability matrix was used as input into hierarchical clustering to reveal patterns recognized by the model.
