# Supplementary figures and images for: A Silent Operon of Photorhabdus luminescens Encodes a Prodrug Mimic of GTP
Source: mBio. 2022 May 16;13(3):e00700-22. doi: 10.1128/mbio.00700-22 (PMC9239236; doi:10.1128/mbio.00700-22)

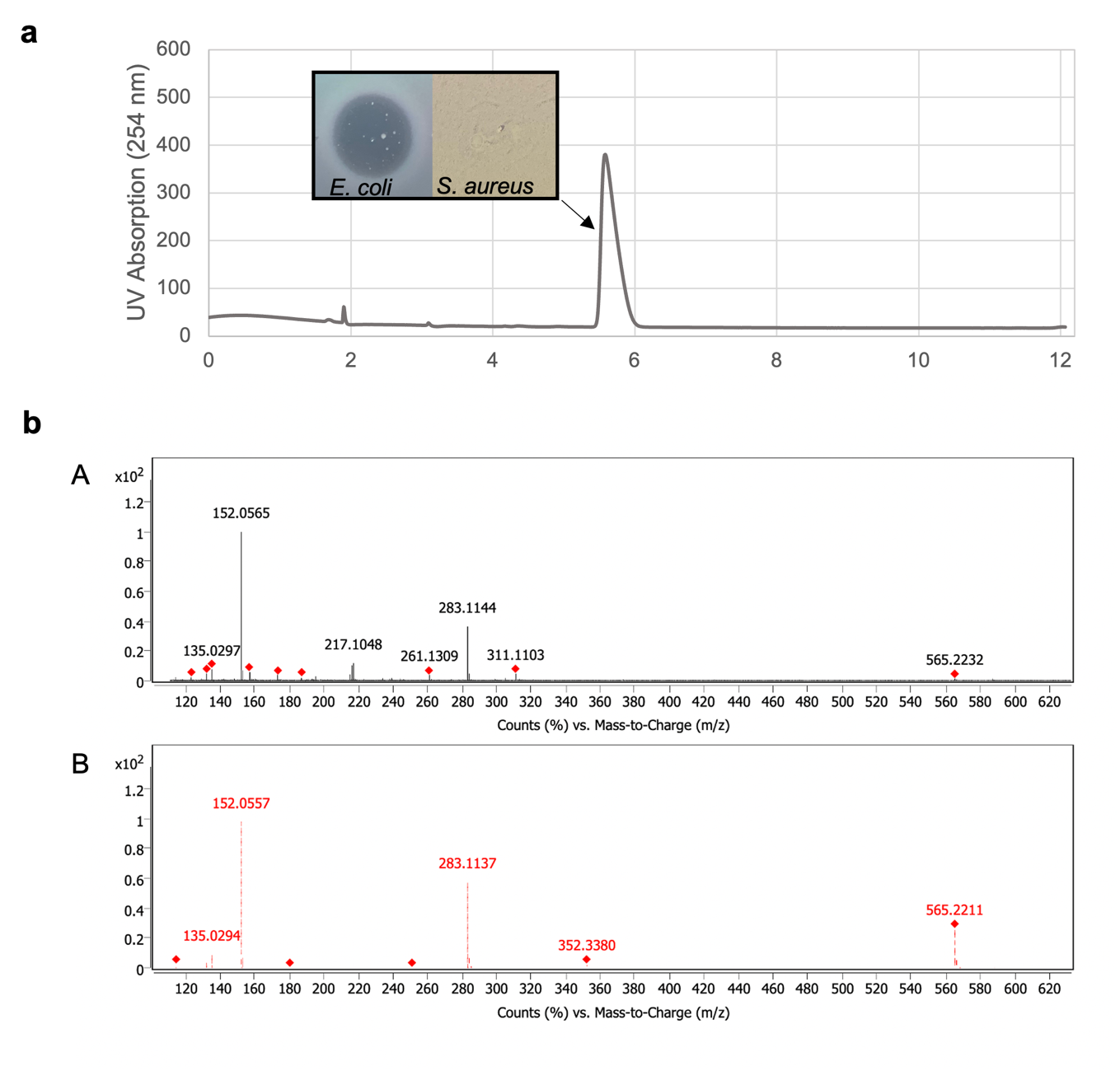

Supplement: FIG S1 [file mbio.00700-22-s0001.docx]

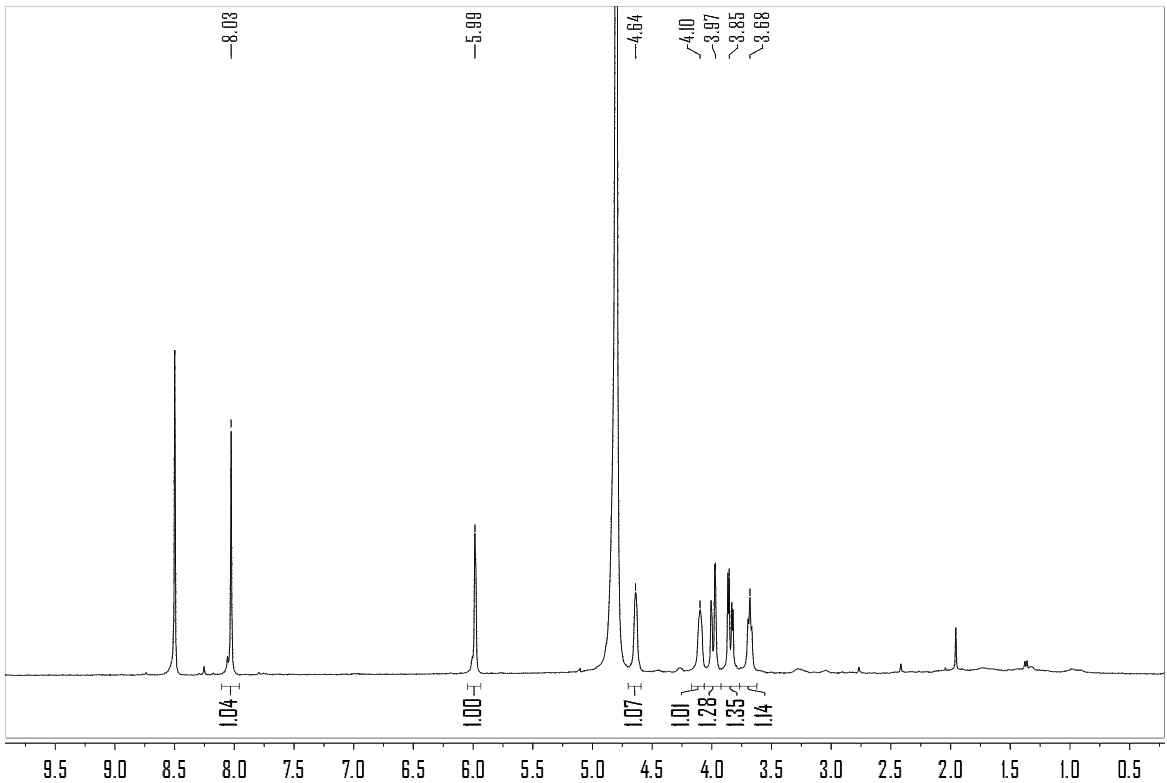

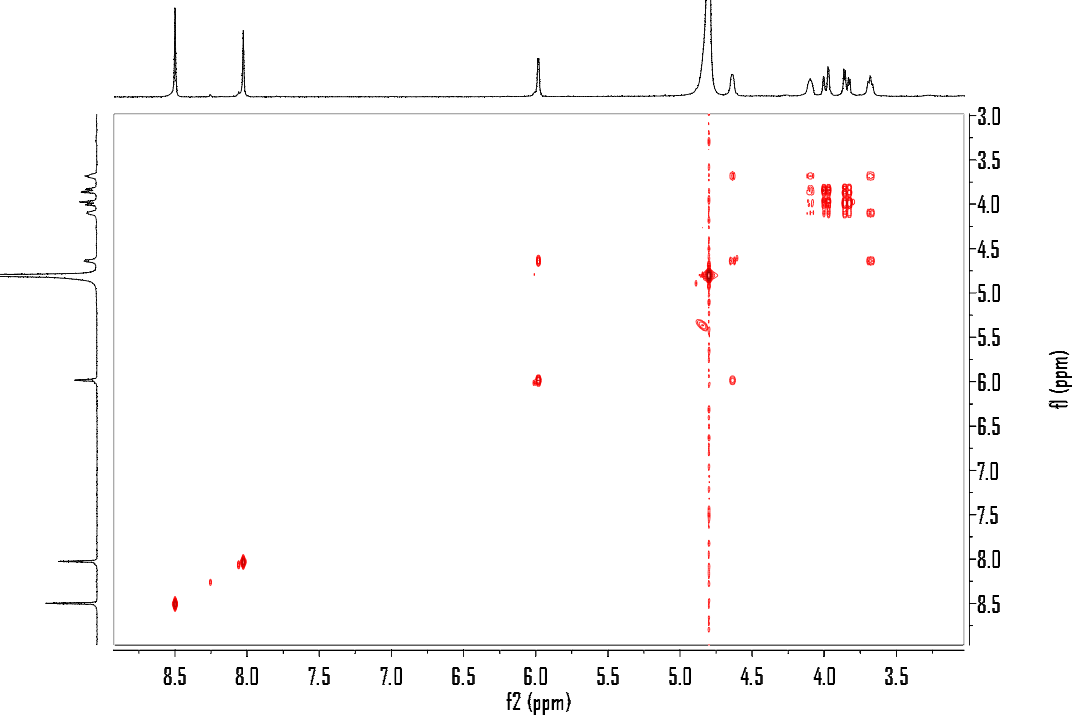


**a**


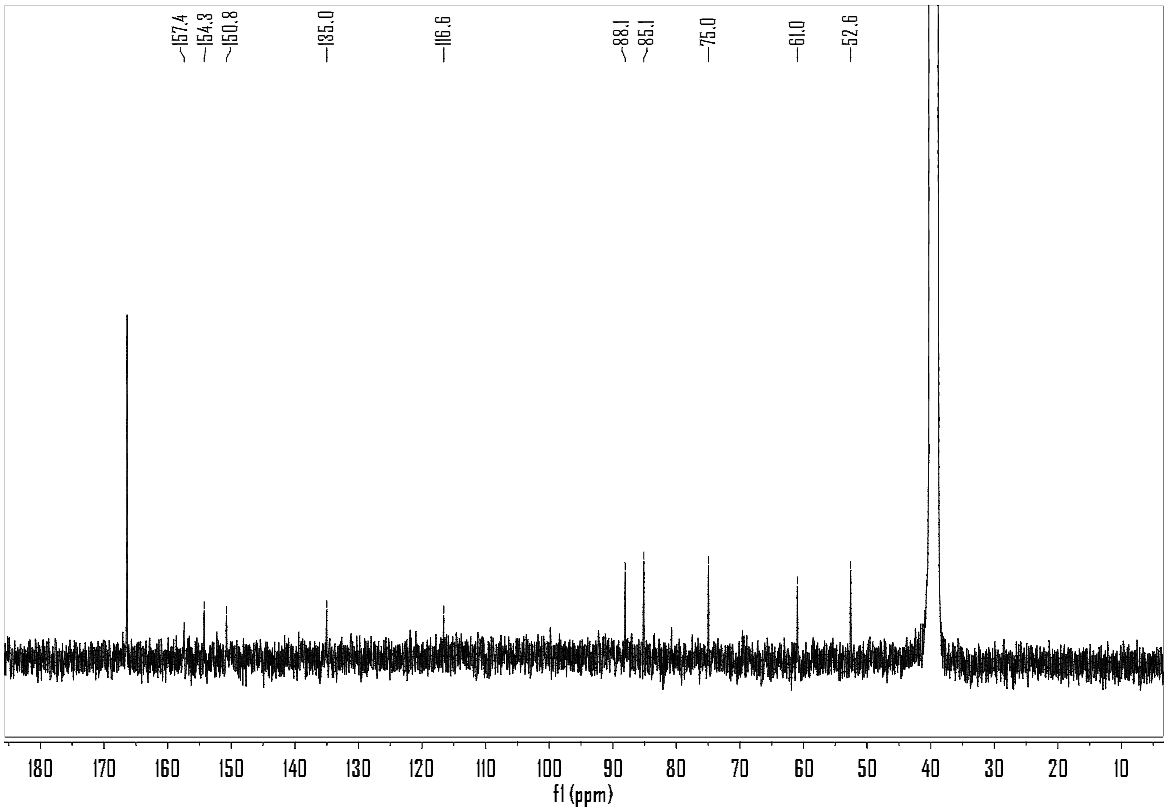


**c**

**d**

**b**

Supplement: FIG S2 [file mbio.00700-22-s0002.docx]

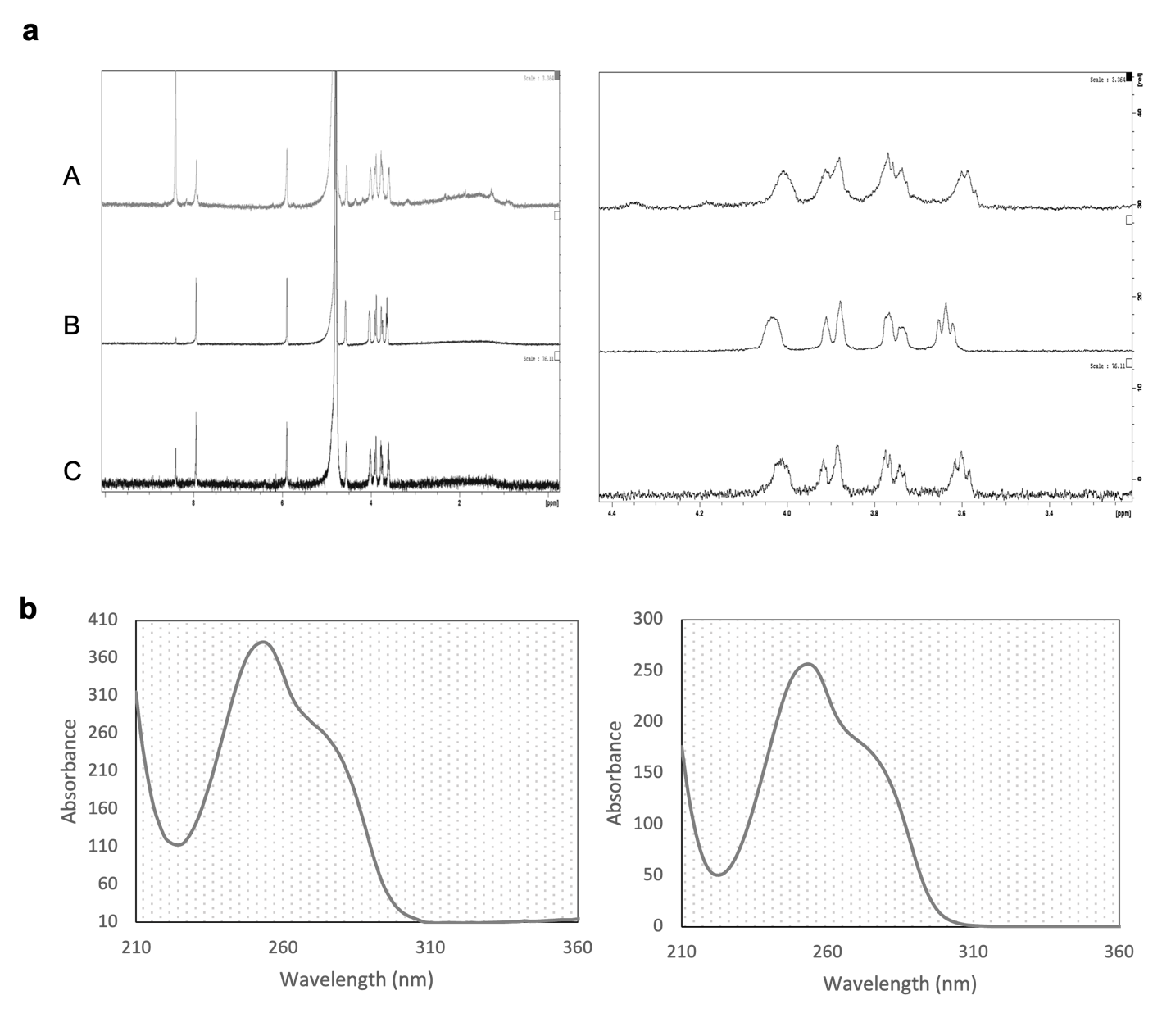

Supplement: FIG S3 [file mbio.00700-22-s0003.docx]

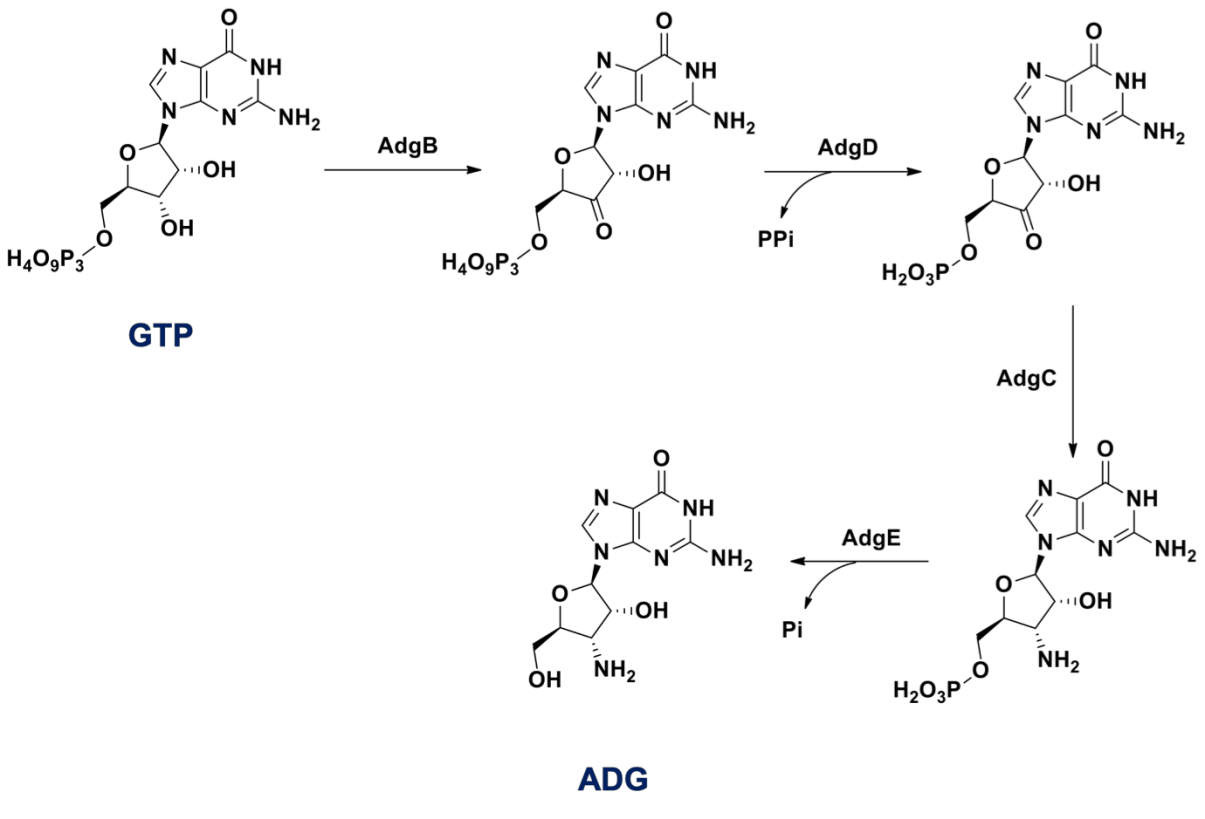

Supplement: FIG S4 [file mbio.00700-22-s0004.docx]

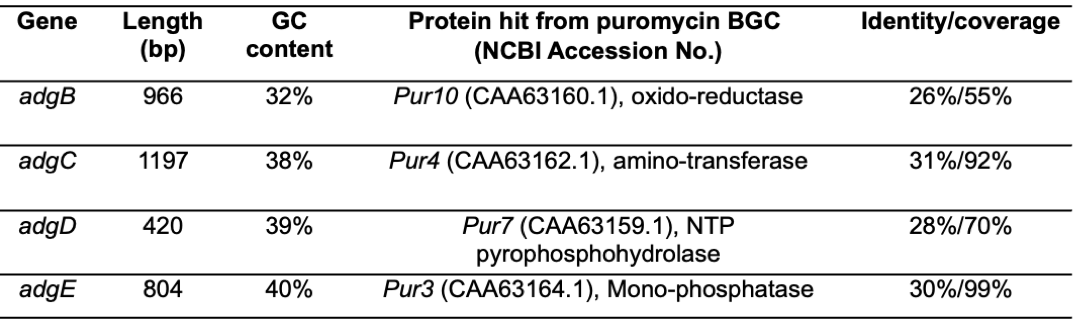

Supplement: TABLE S3 [file mbio.00700-22-s0009.docx]

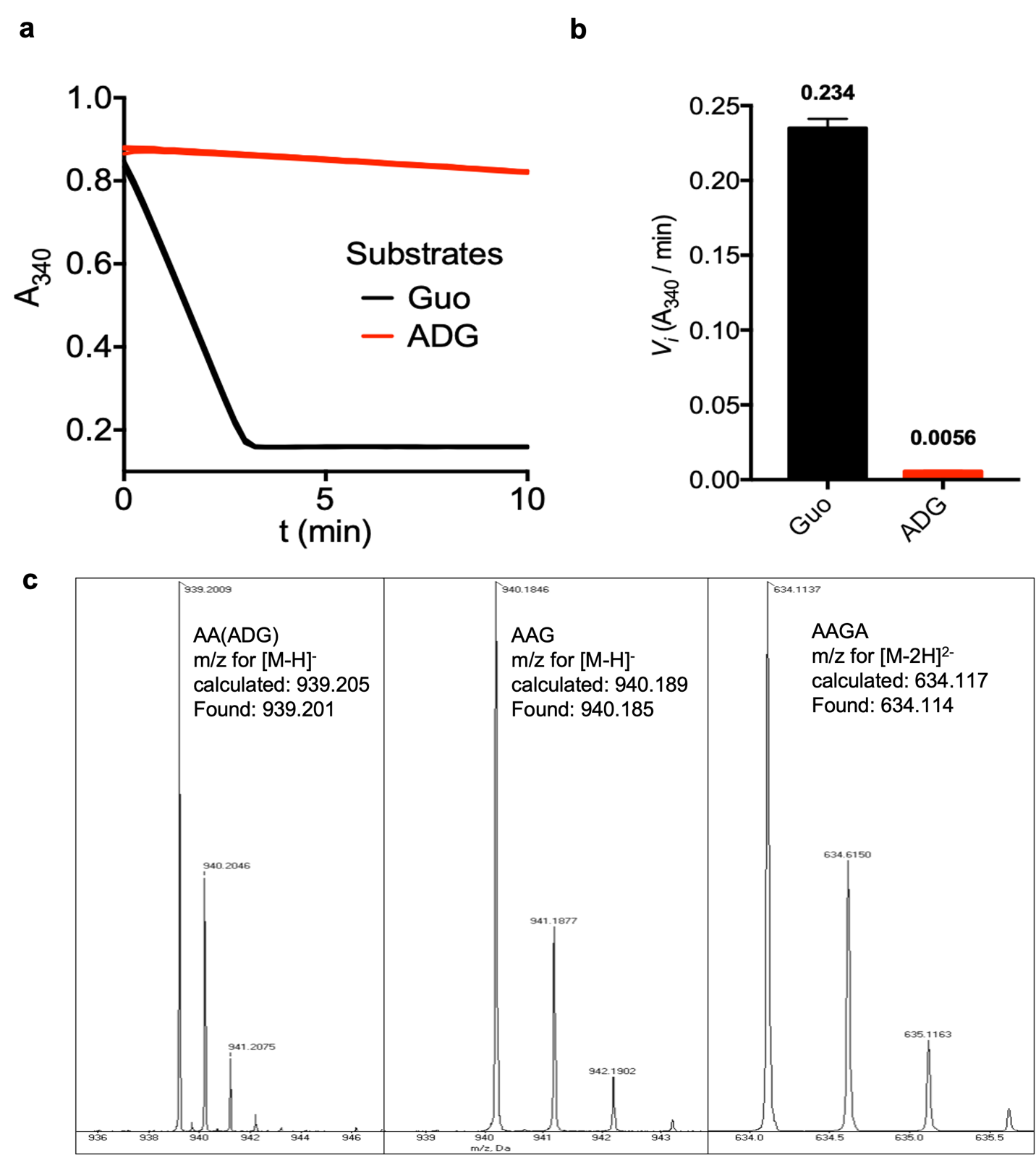

Supplement: FIG S5 [file mbio.00700-22-s0005.docx]

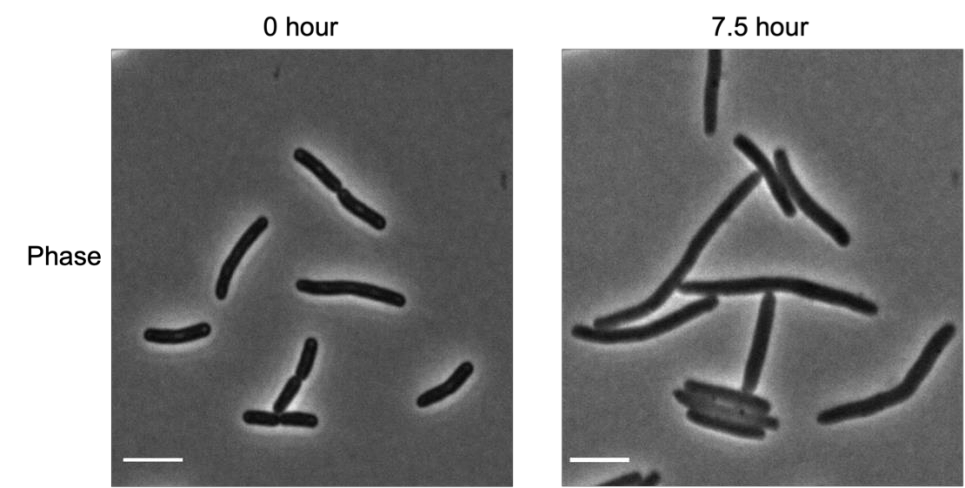

Supplement: FIG S6 [file mbio.00700-22-s0006.docx]

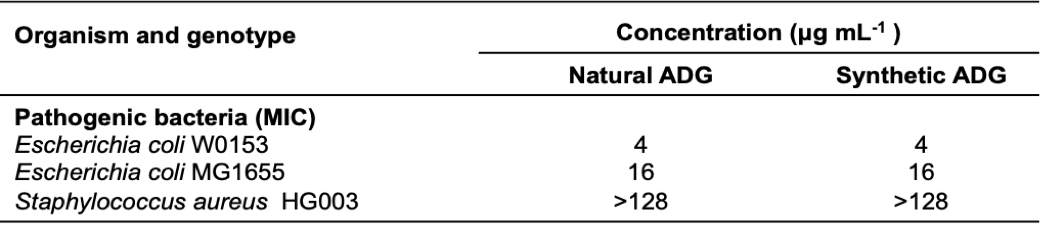

Supplement: TABLE S2 [file mbio.00700-22-s0008.docx]
